# Crystalline silica-induced proinflammatory eicosanoid storm in novel alveolar macrophage model quelled by docosahexaenoic acid supplementation

**DOI:** 10.3389/fimmu.2023.1274147

**Published:** 2023-11-09

**Authors:** Olivia K. Favor, Lichchavi D. Rajasinghe, Kathryn A. Wierenga, Krishna R. Maddipati, Kin Sing Stephen Lee, Andrew J. Olive, James J. Pestka

**Affiliations:** ^1^ Department of Pharmacology and Toxicology, Michigan State University, East Lansing, MI, United States; ^2^ Institute for Integrative Toxicology, Michigan State University, East Lansing, MI, United States; ^3^ Department of Food Science and Human Nutrition, Michigan State University, East Lansing, MI, United States; ^4^ Department of Biochemistry and Molecular Biology, Michigan State University, East Lansing, MI, United States; ^5^ Department of Pathology, Wayne State University, Detroit, MI, United States; ^6^ Department of Chemistry, Michigan State University, East Lansing, MI, United States; ^7^ Department of Microbiology and Molecular Genetics, Michigan State University, East Lansing, MI, United States

**Keywords:** crystalline silica, docosahexaenoic acid, alveolar macrophage, lipidome, oxylipin

## Abstract

**Introduction:**

Phagocytosis of inhaled crystalline silica (cSiO_2_) particles by tissue-resident alveolar macrophages (AMs) initiates generation of proinflammatory eicosanoids derived from the ω-6 polyunsaturated fatty acid (PUFA) arachidonic acid (ARA) that contribute to chronic inflammatory disease in the lung. While supplementation with the ω-3 PUFA docosahexaenoic acid (DHA) may influence injurious cSiO_2_-triggered oxylipin responses, *in vitro* investigation of this hypothesis in physiologically relevant AMs is challenging due to their short-lived nature and low recovery numbers from mouse lungs. To overcome these challenges, we employed fetal liver-derived alveolar-like macrophages (FLAMs), a self-renewing surrogate that is phenotypically representative of primary lung AMs, to discern how DHA influences cSiO_2_-induced eicosanoids.

**Methods:**

We first compared how delivery of 25 µM DHA as ethanolic suspensions or as bovine serum albumin (BSA) complexes to C57BL/6 FLAMs impacts phospholipid fatty acid content. We subsequently treated FLAMs with 25 µM ethanolic DHA or ethanol vehicle (VEH) for 24 h, with or without LPS priming for 2 h, and with or without cSiO_2_ for 1.5 or 4 h and then measured oxylipin production by LC-MS lipidomics targeting for 156 oxylipins. Results were further related to concurrent proinflammatory cytokine production and cell death induction.

**Results:**

DHA delivery as ethanolic suspensions or BSA complexes were similarly effective at increasing ω-3 PUFA content of phospholipids while decreasing the ω-6 PUFA arachidonic acid (ARA) and the ω-9 monounsaturated fatty acid oleic acid. cSiO_2_ time-dependently elicited myriad ARA-derived eicosanoids consisting of prostaglandins, leukotrienes, thromboxanes, and hydroxyeicosatetraenoic acids in unprimed and LPS-primed FLAMs. This cSiO_2_-induced eicosanoid storm was dramatically suppressed in DHA-supplemented FLAMs which instead produced potentially pro-resolving DHA-derived docosanoids. cSiO_2_ elicited marked IL-1α, IL-1β, and TNF-α release after 1.5 and 4 h of cSiO_2_ exposure in LPS-primed FLAMs which was significantly inhibited by DHA. DHA did not affect cSiO_2_-triggered death induction in unprimed FLAMs but modestly enhanced it in LPS-primed FLAMs.

**Discussion:**

FLAMs are amenable to lipidome modulation by DHA which suppresses cSiO_2_-triggered production of ARA-derived eicosanoids and proinflammatory cytokines. FLAMs are a potential *in vitro* alternative to primary AMs for investigating interventions against early toxicant-triggered inflammation in the lung.

## Introduction

Approximately 2.3 million American workers are exposed to cSiO_2_ levels that exceed the Occupational Health and Safety Administration’s (OSHA’s) Permissible Exposure Limits (1), with the highest exposure levels in construction, manufacturing, sandblasting, farming, ceramics, and dentistry work (2, 3). Occupational exposure to respirable crystalline silica (cSiO_2_) is etiologically linked to silicosis, lung cancer, and systemic autoimmune disease (4–7). When inhaled into the lung, cSiO_2_ particles travel to the alveoli where they are readily phagocytosed by alveolar macrophages (AMs) (8). Following phagocytosis, cSiO_2_ induces lysosomal membrane permeabilization (LMP), mitochondrial toxicity, reactive oxygen species (ROS) formation, NLRP3 inflammasome activation, oxylipin generation, release of proinflammatory proteins, and cell death (9, 10). The resultant bioactive oxylipins derived from the 20-carbon ω-6 polyunsaturated fatty acid (PUFA) arachidonic acid (ARA) (i.e., eicosanoids) and proinflammatory cytokines can act in a paracrine manner to induce expression and secretion of additional proinflammatory mediators by neighboring AMs and other cells. If not properly cleared, cSiO_2_ particles released from dead AMs can again be taken up by viable AMs or recruited macrophages, contributing to a perpetual cycle of inflammation, cell death, and tissue damage. Accordingly, tissue-resident AMs play a critical role in the initiation of persistent cSiO_2_-triggered lung inflammation and development of chronic disease.

Relative to eicosanoids, it is well-known that inflammatory stimuli in the lung can activate phospholipase A2 (PLA2) in macrophages which promotes release of ARA, one of the most abundant PUFAs in the sn-2 position of membrane phospholipids, into the cytosol. Free ARA is then converted into prostaglandins (PGs), leukotrienes (LTs), thromboxanes (TXs), and hydroxyeicosatetraenoic acids (HETEs) (11, 12). Some of these eicosanoids promote further immune cell infiltration and have been associated with damage to pulmonary tissue and increased symptom severity in many lung diseases including COVID-19, asthma, cystic fibrosis, chronic obstructive pulmonary disease (COPD), and extrinsic allergic alveolitis (13–17). Importantly, several previous studies have reported that exposing rat, bovine, and human AMs to cSiO_2_ drives biosynthesis of classical ARA-derived eicosanoids, such as PGE2, LTB4, TXB2, and 5-HETE (18–21), some of which have been recently described in human patients with silicosis and in a preclinical C57BL/6 mouse silicosis model (22). The possibility exists that many more oxylipins are elicited by AMs in response to the particle, perhaps tantamount to an “eicosanoid storm” (13–17), that contribute as a group to the potent inflammatory effects of the particle in the lung.

One potential intervention against cSiO_2_-induced ARA-derived eicosanoid generation and downstream lung inflammation is dietary supplementation with ω-3 polyunsaturated fatty acids (PUFAs) such as docosahexaenoic acid (C22:6, ω-3, DHA) (23, 24). ω-3 PUFAs can prevent excessive inflammatory responses and promote pro-resolving immune responses by displacing ω-6 PUFAs from the plasma membrane and i) competing with ω-6 PUFAs as substrates for cyclooxygenase (COX), lipoxygenase (LOX) and cytochrome P450 (CYP450) enzymes thereby reducing production of proinflammatory eicosoanoids while increasing potentially pro-resolving DHA-derived oxylipins (i.e., docosanoids), ii) altering lipid raft structures and attenuating downstream signal transduction, iii) preventing NF-κB-driven expression of proinflammatory mediators, and iv) enhancing efferocytosis of cell corpses by phagocytes (25–28). In both mice and humans, DHA consumption leads to decreased tissue and plasma levels of ARA and related eicosanoids concurrently with increased DHA and related docosanoids (24, 29–33). It is thus of great interest to understand how skewing AM membrane content from ω-6 PUFAs to ω-3 PUFAs influences the cSiO_2_-induced oxylipin profile, proinflammatory cytokine production, and cell death induction. As a first step toward that goal, we have demonstrated in RAW264.7 murine macrophages transfected with apoptosis-associated speck-like protein containing a caspase recruitment domain (ASC) that DHA displaces ω-6 ARA and ω-9 oleic acid (OA) from membrane phospholipids and suppresses cSiO_2_-induced NLRP3 inflammasome activation and IL-1 release (27). However, neither transformed macrophage cell lines (e.g. RAW264.7 or THP-1 cells) nor other widely used primary macrophages (e.g., bone marrow-derived macrophages [BMDMs] or peritoneal macrophages) adequately model the tissue-specific phenotype of primary AMs (34). Specifically, BMDMs originate from hematopoetic stem cells while peritoneal macrophages and AMs originate from erythromyeloid progenitor cells in the fetal liver (35). In addition, BMDMs express high CD115 and Ly6G (36) and peritoneal macrophages express high B7-H1 and CD11b (36, 37), whereas AMs express low CD11b and high CD11c, SiglecF, and Ly6G (38). Thus, there is a need to conduct these studies in primary AMs or a more physiologically relevant model.

Several barriers hinder mechanistic research efforts into primary AM function. Since only approximately 1-5 × 10^5^ primary cells are typically recovered from one adult mouse by bronchoalveolar lavage, obtaining sufficient numbers of cells for rigorous *in vitro* investigations requires sacrificing large numbers of mice (39). Additionally, primary AMs may undergo phenotypic changes upon being cultured and therefore might not completely reflect *in vivo* AM function (40–42). Recently, our laboratory has overcome barriers to AM culture by developing an *ex vivo* fetal liver-derived alveolar-like macrophage (FLAM) model that is non-transformed and self-replicating in the presence of GM-CSF and TGF-β (43). Like primary AMs, FLAMs are characterized by high surface expression of SiglecF and CD11c, low surface expression of CD14, and stable expression of AM-specific genes such as *Marco*. In addition, the kinetics of cSiO_2_ phagocytosis, cell death, and IL-1 cytokine release in FLAMs mirrors that of primary AMs. Accordingly, FLAMs are convenient, phenotypically relevant AM surrogates for conducting thorough *in vitro* mechanistic studies involving cSiO_2_ exposure and the effects of ω-3 intervention.

Here, we hypothesized that DHA would inhibit proinflammatory eicosanoid production induced by cSiO_2_. To test our hypothesis, we employed FLAMs as an AM model and targeted LC-MS lipidomics to understand how DHA incorporation influences cSiO_2_-induced oxylipin generation within 4 h and further related these results to proinflammatory cytokine production and induction of cell death over the same time window. Our findings suggest FLAMs to be a promising *in vitro* alternative to primary AMs for future investigations of how modulation of their cellular lipidome influences eicosanoid production by cSiO_2_ and how these differential responses are potentially linked to cSiO_2_-induced inflammation in the lung.

## Materials and methods

### Key reagents

All key reagents used in this study and their corresponding catalog numbers are outlined in **Supplementary Table 1**.

### Fetal liver-derived alveolar macrophage isolation and cell culture

Experimental protocols were approved by the Institutional Animal Care and Use Committee at Michigan State University (MSU) (Animal Use Form [AUF] #PROTO201800113) in accordance with guidelines established by the National Institutes of Health. Six- to 8-wk-old C57BL/6 mice (cat. #000664) were procured from the Jackson Laboratory (Bar Harbor, ME) and were given freely accessible food and water. Animal facilities were maintained with a 12 h light/dark cycle at consistent temperature (21-24°C) and humidity (40-55%). Mice were bred for FLAM isolation as previously described (43, 44). At 14-18 gestational days, pregnant dams (8-10 wk of age) were euthanized by CO_2_ asphyxiation for 10 min to ensure death to the neonatal mice (14-18 gestational days), which are resistant to anoxia. As a secondary method of euthanasia, cervical dislocation was performed on the dam and blood supply was cut off to the fetuses. Upon removing the fetuses from the dam, fetal livers were carefully removed and dissociated in ice-cold DPBS^-/-^ (DPBS without calcium and magnesium) by gentle pipetting. Liver cells were pelleted by centrifugation at 220 *x g* for 5 min at 4°C, resuspended in complete FLAM medium (RPMI 1640 medium, 10% fetal bovine serum, 1% penicillin-streptomycin, 30 ng/ml recombinant mouse GM-CSF, and 20 ng/ml recombinant human TGF-β1), seeded in 100 mm tissue-culture treated dishes, and incubated at 37°C and 5% CO_2_. Medium was changed every 1-2 d and non-adherent dead cells discarded. Adherent fetal liver monocytes were lifted with DPBS^-/-^ containing 10 mM EDTA and gentle scraping upon reaching 70-90% confluency. After 1-2 wk of culture, cells developed a round morphology resemblant of AMs and were frozen for future use. Mature, differentiated FLAMs were routinely confirmed to be CD14^+^ low, SiglecF^+^ high, and CD11c^+^ high by flow cytometry as previously described (43). Cells between passage 5 and 10 were used for this study.

### Comparative effects of DHA delivery using ethanolic suspensions and bovine serum albumin complexes on phospholipid fatty acid profile in FLAMs

FLAMs were seeded in 6-well plates at a density of 4.50×10^5^ cells/well in complete FLAM medium. Cells were incubated overnight to achieve 70-90% confluency before beginning treatments. The next day, cells were washed once with sterile DPBS^-/-^, and were then incubated for 24 h in fresh complete FLAM medium containing either 1) ethanolic DHA (25 µM) or ethanol (EtOH) vehicle (VEH), or 2) 25 µM DHA complexed to BSA at a 3:1 ratio as previously described (27, 28) or 8.33 µM BSA VEH. Following treatments, FLAMs were pelleted and stored in 100% methanol at -80°C before total fatty acids were measured at OmegaQuant Inc. using gas chromatography (GC) with flame ionization detection as previously described (45).

A standard mixture of fatty acids (GLC OQ-A; NuChek Prep, Elysian, MN) was used to generate calibration curves for individual fatty acids and identify fatty acids present in each sample. The following 24 fatty acids were identified (by class): i) saturated (14:0, 16:0, 18:0, 20:0, 22:0, 24:0), ii) cis monounsaturated (16:1, 18:1, 20:1, 24:1), iii) cis ω-6 polyunsaturated (18:2ω6, 18:3ω6, 20:2ω6, 20:3ω6, 20:4ω6, 22:4ω6, 22:5ω6), and iv) cis ω-3 polyunsaturated (18:3ω3, 20:5ω3, 22:5ω3, 22:6ω3). Proportions of individual fatty acids in each sample were expressed as a percentage of total identified fatty acids.

Two biomarkers of ω-3 tissue incorporation, percent ω-3 PUFAs and highly unsaturated fatty acid (HUFA) score, were calculated as previously described (23) using the following equations, with total fatty acids (FA) equaling the sum of all analyzed FA and total HUFA equaling the sum of dihomo-γ-linolenic acid (DGLA; 20:3ω6), ARA (20:4ω6), eicosatetraenoic acid (EPA; 20:5ω3), docosatetraenoic acid (DTA; 22:4ω6), ω-6 docosapentaenoic acid (DPA; 22:5ω6), ω-3 DPA (22:5ω3), and DHA (22:6ω3):


$$\text{Percent ω}-3\text{ PUFAs}=\frac{EPA+DHA}{Total\;FA}\times 100\%$$

$$\text{ω}-3\;HUFA\;score=\frac{EPA+DPA_{\text{ω}-3}+DHA}{Total\;HUFA}\times 100\%$$

### Determination of DHA’s effects on cSiO_2_-induced generation of oxylipins in FLAMs by targeted LC-MS lipidomics

For lipidomics analyses, FLAMs were seeded in 6-well plates at a density of 4.50×10^5^ cells/well in complete FLAM medium. Cells were incubated overnight to achieve 70-90% confluency before beginning treatments. The next day, cells washed once with sterile DPBS^-/-^, fresh complete FLAM medium containing either 25 µM ethanolic DHA or EtOH VEH, then cells incubated for 24 h. Following DHA or VEH treatment, cells were washed once with sterile DPBS^-/-^, treated with either 20 ng/ml LPS in DPBS^-/-^ or DPBS^-/-^ VEH in DPBS^+/+^ (DPBS containing calcium and magnesium) for 2 h, then exposed to 12.5 µg/cm^2^ cSiO_2_ or DPBS^-/-^ VEH for 1.5 or 4 h. These timepoints were selected for cSiO_2_ exposure to assess acute toxic effects of the particle on FLAMs instead of prolonged effects. LPS and cSiO_2_ exposures were done in DPBS^+/+^ to minimize interference from fatty acids present in cell culture medium during oxylipin analyses. Each treatment condition was tested using three biological replicates and one technical replicate.

Cell culture supernatants and cells were collected at 0, 1.5, and 4 h after cSiO**_2_** treatment and pooled together prior to lipidomic analysis. Following treatments, ice-cold methanol was added to each well, resulting in a final sample volume of 3 ml (1 ml cell culture supernatant + 2 ml methanol). To each sample, 60 µl of antioxidant cocktail (0.2 mg/ml butylated hydroxytoluene, 0.2 mg/ml triphenylphosphine, 0.6 mg/ml EDTA) was added to achieve a total cocktail concentration of 5% (v/v) (46). After subjecting the cells and supernatant to centrifugation at 0°C and 10,000 *x g* to separate the cellular debris, the resulting supernatant was stored in tubes at -80°C under nitrogen gas. Cells and supernatants within each well were pooled together, then samples were frozen at -80°C until liquid chromatography-mass spectrometry (LC-MS) analysis. Targeted LC-MS lipidomics for 156 lipid metabolites was conducted at the Lipidomics Core Facility at Wayne State University as previously described (47–49). Briefly, 100 µl aliquots of cellular samples were thawed and spiked with a cocktail of deuterated internal standards (5 ng each of PGE_1_-d_4_, RvD2-d_5_, LTB_4_-d_4_, and 15[S]-HETE-d_8_; Cayman Chemical, Ann Arbor, MI) for quantification of oxylipins and recovery. Then, oxylipins were extracted by using C18 extraction columns that were washed with 15% (v/v) methanol and subsequently hexane, dried in a vacuum, eluted with methanol containing 0.1% (v/v) formic acid, dried under nitrogen gas, and dissolved in a 1:1 mixture of methanol:25 mM aqueous ammonium acetate. Extracted oxylipins were subjected to high-performance liquid chromatography (HPLC) using a Luna C18 (3 µm, 2.1×150 mm) column connected to a Prominence XR system (Shimadzu, Somerset, NJ) then analyzed with a QTrap5500 mass spectrometer (AB Sciex, Singapore) set to negative ion mode. Analyst 1.6 software (AB Sciex) and MultiQuant software (AB Sciex) were used to collect and quantify the data in units of ng, respectively. Lipid metabolite classifications are provided in **Supplementary Table 2**, and mass spectra of representative metabolites are given in reference (50).

### Assessment of DHA’s effects on cSiO_2_-induced release of lysosomal cathepsins, LDH, and proinflammatory cytokines in FLAMs

FLAMs were seeded in 24-well plates at a density of 1.50×10^5^ cells/well in complete FLAM medium and then cultured with DHA or VEH, LPS or VEH, and cSiO_2_ or VEH under the conditions described for the lipidomics study. LPS and cSiO_2_ exposures were done in DPBS^+/+^ to maintain consistent experimental conditions between oxylipin analyses and other analyses conducted in this study. Cell culture supernatants were collected by centrifugation at 200 *x g* at 0, 1.5, and 4 h post cSiO_2_ exposure and analyzed for proinflammatory cytokines, cathepsin B activity, and lactate dehydrogenase (LDH) release.

Cathepsin B activity was determined using a fluorescent assay as previously described (51). Briefly, 50 µl of cell culture supernatant and 2 µg Z-LR-AMC were combined in 96-well plates and adjusted to a final volume of 150 µl/well, then all samples were incubated for 1 h at 37°C. Sample fluorescence was then measured using a FilterMax F3 Multimode plate reader (Molecular Devices, San Jose, CA) set to 380 nm excitation and 460 nm emission. Cathepsin B activity in each well was calculated in units of relative fluorescence units (RFU) by the following equation:


$$RFU_{sample}-RFU_{sample\;blank}=cathepsin\;B\;activity$$

LDH activity was measured as previously described (27, 28). Separate wells of untreated FLAMs were included and designated as max-kill (MK) samples and incubated with 0.2% Triton-X (Millipore Sigma) for 5 min. After all treatments, supernatants were collected from all wells and 50 µl was transferred to non-treated, flat-bottom 96-well plates in duplicate. DPBS^+/+^ was used as a sample blank, and DPBS^+/+^ containing 0.2% (v/v) Triton-X was used as a MK blank. 100 µl of LDH assay reagent (15 µM 1-methoxyphenazine methosulfate [PMS], 2 mM iodonitrotetrazolium [INT], 3.2 mM β-nicotinamide adenine dinucleotide [NAD] sodium salt, and 160 mM lithium lactate in 0.2 M Tris-HCl, pH 8.2) was added to each well, and assay plates were incubated at room temperature for 15 min in the dark. Sample absorbance was then measured using a FilterMax F3 Multimode plate reader (Molecular Devices, San Jose, CA) set to a wavelength of 492 nm. Percent cell death in each well was calculated by the following equation:


$$\frac{Sample_{abs}-Sample\;Blank_{abs}}{Max\;Kill_{abs}-Max\;Kill\;Blank_{abs}}\times 100\%=Percent\;Cell\;Death$$

Concentrations of proinflammatory cytokines (i.e., IL-1α, IL-1β, TNF-α) were quantified by enzyme-linked immunosorbent assay (ELISA) using corresponding mouse R&D Systems DuoSet kits according to the manufacturer’s instructions.

### Determination of DHA effects on cSiO_2_-induced lysosomal membrane permeabilization, mitochondrial depolarization, and cell death in FLAMs using live-cell microscopic imaging

FLAMs were seeded in 48-well plates 0.625×10^5^ cells/well in complete FLAM medium to achieve 50% confluency after overnight incubation. The next day, cells were washed once with sterile DPBS^-/-^ then treated with either 25 µM ethanolic DHA or EtOH VEH as a control in complete FLAM medium. After 24 h, cells were washed once with sterile DPBS^-/-^ and primed with 20 ng/ml LPS or PBS VEH for 1.5 h. Following LPS priming, cells were washed with sterile DPBS^+/+^ then subsequently incubated for 30 min at 37°C in the dark with 50 nM LysoTracker Red DND-99 in DPBS^+/+^ to label lysosomes, 25 nM MitoTracker Red CMXRos to label mitochondria, or 200 nM SYTOX Green to detect cell death. All fluorescent dyes were diluted in DPBS^+/+^ prior to addition to the cells, and 20 ng/ml LPS was simultaneously added to appropriate wells to allow 2 h of total LPS priming prior to cSiO_2_ treatment. After 30 min of cell staining, a freshly prepared cSiO_2_ stock suspension was added dropwise to a final concentration of 0 or 12.5 µg/cm^2^. LPS and cSiO_2_ exposures were done in DPBS^+/+^ to maintain consistent experimental conditions between oxylipin analyses and other analyses conducted in this study.

Live-cell fluorescence microscopy began immediately after adding cSiO_2_ to the cells. Images of live cells were taken at 0, 1.5, and 4 h post cSiO_2_ treatment using an EVOS FL Auto 2 Cell Imaging System (ThermoFisher Scientific) with an onstage, temperature-controlled incubator. Each experimental condition was tested using 3 biological replicates. For each well, 2-4 images were acquired using fields of view defined before the beginning of the experiment. LysoTracker Red and MitoTracker Red were detected using the Texas Red light cube (Ex: 585/29 nm, Em: 628/32 nm), and SYTOX Green was detected using the GFP light cube (Ex: 482/25 nm, Em: 524/24 nm).

Acquired images were analyzed for lysosomal integrity, mitochondrial integrity, and cell death using CellProfiler 4.2.1 as previously described (43, 52). Briefly, lysosomal integrity, mitochondrial integrity, and cell death were assessed by quantifying the number of LysoTracker Red^+^, MitoTracker Red^+^, and SYTOX Green^+^ cells, respectively. LysoTracker Red^+^ and MitoTracker Red^+^ puncta were omitted if fluorescent intensity fell below preset minimum thresholds, which were chosen to omit false positives quantified from background fluorescence. Raw counts of LysoTracker Red^+^, MitoTracker Red^+^, and SYTOX Green^+^ cells were further analyzed using RStudio 2022.07.1 + 554 (Posit, Boston, MA).

### Data analysis and statistics

For oxylipin data, MetaboAnalyst Version 5.0 (Xia Lab, Quebec, Canada, www.metaboanalyst.ca/) (53) was used to conduct statistical analyses. First, raw ng values were converted to corresponding pmol values in Microsoft Excel (50). Then, in MetaboAnalyst, the one factor statistical analysis module was chosen, and data were uploaded as a comma separated values (.csv) file with samples in unpaired columns and features (i.e., metabolites) in rows. Features with >70% missing data were removed from the dataset, and remaining missing values were estimated by replacing with the corresponding limits of detection (LODs; 1/5 of the minimum positive value of each variable). After the data was cleaned, the data was normalized by auto scaling only, then the data editor option was used to select experimental groups of interest to compare. For comparisons between experimental groups, one-way analysis of variance (ANOVA) (FDR = 0.05) followed by Tukey’s honestly significant difference (HSD) *post-hoc* test was used, with FDR q<0.05 considered statistically significant. Asterisks (*) indicate statistically significant differences (FDR q<0.05) for cSiO_2_-treated groups and their corresponding non-cSiO_2_-treated controls. Hashes (#) indicate statistically significant differences (FDR q<0.05) for DHA-treated groups and their corresponding non-DHA-treated controls. Crosses (†) indicate statistically significant differences (FDR q<0.05) for LPS-treated groups and their corresponding non-LPS-treated controls.

For all other endpoints, GraphPad Prism Version 9 (GraphPad Software, San Diego, CA, www.graphpad.com) was used to conduct statistical analyses. The ROUT outlier test (Q = 1%) and the Shapiro-Wilk test (p<0.01) were used to identify outliers and assess normality in the data, respectively. For comparisons between two experimental groups, non-normal and semiquantitative data were analyzed by the Mann-Whitney nonparametric test. The F test (p<0.05) was used to test the assumption of equal variances across both groups. Normal data with unequal variances were analyzed using an unpaired t test with Welch’s correction. Normal data that met the assumption of equal variance were analyzed using an unpaired t test. For comparisons between more than two experimental groups, non-normal and semi-quantitative data were analyzed by the Kruskal-Wallis nonparametric test followed by Dunn’s *post-hoc* test. The Brown-Forsythe test (p<0.01) was used to test the assumption of equal variances across treatment groups. Normal data with unequal variances were analyzed using the Brown-Forsythe/Welch analysis of variance (ANOVA) followed by Dunnett’s T3 *post-hoc* test. Normal data that met the assumption of equal variance were analyzed by standard one-way ANOVA followed by Tukey’s *post-hoc* test. Data are presented as mean ± standard error of the mean (SEM), with a p-value < 0.05 considered statistically significant.

## Results

### DHA displaces the ω-9 monounsaturated fatty acid oleic acid and the ω-6 polyunsaturated fatty acid arachidonic acid from membrane phospholipids in FLAMs

**Figure 1:**
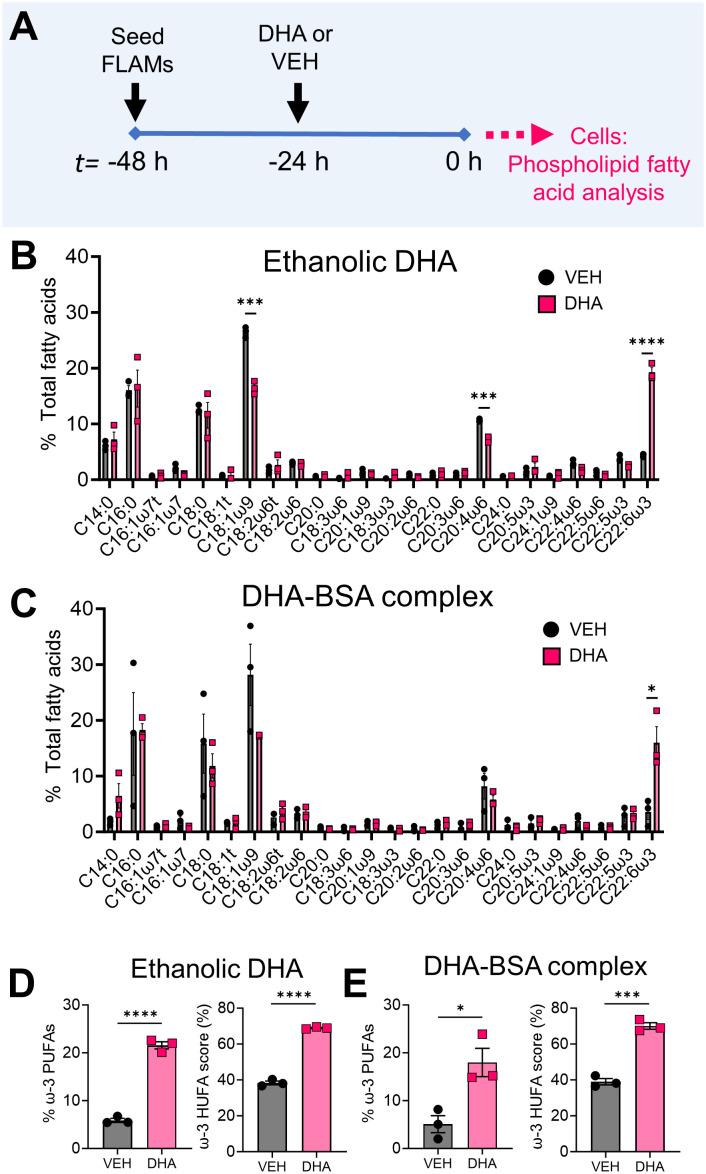
Supplementation of FLAMs with DHA significantly decreases oleic acid (OA) and arachidonic acid (ARA) content in FLAMs. **(A)** FLAMs were treated with i) 25 µM DHA as an ethanolic suspension or ethanol vehicle (VEH) or ii) 25 µM DHA as a BSA complex or 8.33 µM BSA VEH. After 24 h, cells were collected for membrane phospholipid fatty acid analysis by gas-chromatography (GC). **(B, C)** Following treatment with **(B)** ethanolic DHA and **(C)** DHA-BSA complexes, DHA (22:6ω3) displaces ω-9 OA (18:1ω9) and ω-6 ARA (20:4ω6) from FLAMs. **(D, E)** Percent ω-3 PUFAs (i.e., sum of EPA and DHA as a percentage of total fatty acids) and ω-3 highly unsaturated fatty acid (HUFA) score (i.e., sum of EPA, ω-3 DPA, and DHA as a percentage of the sum of 20:3ω6, 20:4ω6, 20:5ω3, 22:4ω6, 22:5ω6, 22:5ω3, and 22:6ω3) are elevated in FLAMs treated with **(D)** ethanolic DHA and **(E)** DHA-BSA complexes. Data are shown as mean ± SEM. *p<0.05, ***p<0.001, ****p<0.0001: Statistically significant differences between VEH-treated FLAMs and DHA-treated FLAMs.

Two major methods for introducing ω-3 PUFAs to cell cultures, ethanolic suspensions and BSA complexes (54), were evaluated for their suitability for incorporating DHA into FLAMs as shown in **Figure 1A**. Ethanolic DHA-treated FLAMs had significantly greater DHA content (19.3% total fatty acids) compared to EtOH VEH-treated FLAMs (4.4% total fatty acids) (**Figure 1B**). Corresponding with these findings, the ω-9 monounsaturated fatty acid (MUFA) oleic acid (OA), the main fatty acid found in fetal bovine serum (55), and the ω-6 PUFA arachidonic acid (ARA), the major precursor PUFA for eicosanoid biosynthesis, were significantly decreased in DHA-treated FLAMs (16.5% and 7.2% total fatty acids, respectively) compared to VEH-treated FLAMs (26.5% and 10.6% total fatty acids, respectively). No notable changes were found for other saturated and unsaturated fatty acids that were analyzed. FLAMs incubated with DHA-BSA complexes or BSA VEH displayed similar DHA membrane incorporation at the expense of OA and ARA (**Figure 1C**) to that seen for ethanolic DHA (**Figure 1B**).

Total fatty acid findings were related to two biomarkers: i) percent ω-3 fatty acids, which is the sum of eicosapentaenoic acid (EPA) and DHA as a percentage of all measured FA, and ii) ω-3 HUFA score, which equals the sum of ω-3 HUFAs (i.e., EPA, ω-3 DPA, and DHA) as a percentage of all measured ω-3/6 HUFAs (i.e., 20:3ω6, 20:4ω6, 20:5ω3, 22:4ω6, 22:5ω6, 22:5ω3, and 22:6ω3) (23). In FLAMs treated with ethanolic DHA, percent ω-3 fatty acids was 22%, while in VEH-treated FLAMs it was 6% (**Figure 1D**). FLAMs treated with ethanolic DHA also demonstrated a comparatively higher ω-3 HUFA score (69%) compared to VEH-treated FLAMs (38%). Similarly, FLAMs treated with DHA-BSA complexes exhibited significant increases in percent ω-3 fatty acids (18%) and ω-3 HUFA score (70%) compared to VEH-treated cells (5% and 39%, respectively) (**Figure 1E**). Based on its relative simplicity, ethanolic DHA delivery was used for all subsequent studies.

### cSiO_2_ and DHA differentially impact production of ω-6 ARA-derived oxylipins and ω-3 DHA-/EPA-derived oxylipins in unprimed and LPS-primed FLAMs

**Figure 2:**
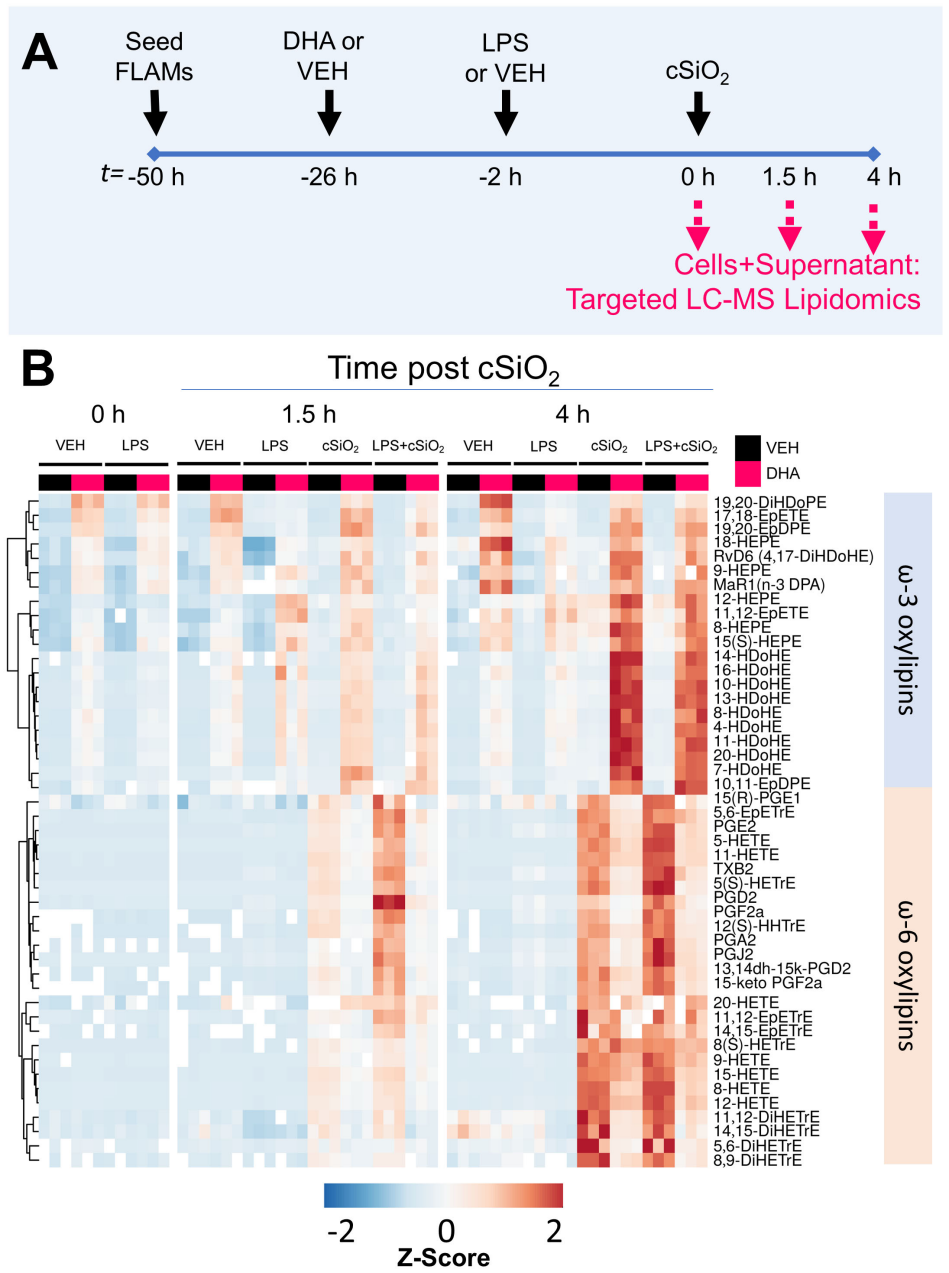
LPS, cSiO_2_, and DHA differentially impact generation of ω-3 and ω-6 oxylipins from FLAMs. **(A)** FLAMs were treated with ethanolic DHA (25 µM) or ethanol vehicle (VEH) for 24 h, primed with LPS (20 ng/ml), and/or exposed to cSiO_2_ (12.5 µg/cm^2^). Cultured FLAMs and supernatants were pooled at t = 0 h, 1.5 h, and 4 h post cSiO_2_ and 156 oxylipins profiled by targeted LC-MS. Treatment conditions were tested using three biological replicates, and oxylipins were measured using one technical replicate per sample. **(B)** Heat maps depicting the concentration of scaled ω-3 and ω-6 oxylipins, using unsupervised clustering with the Euclidean distance method. As an exception, 5(S)-HETrE is derived from ω-9 mead acid (C20:3ω9).

**Figure 3:**
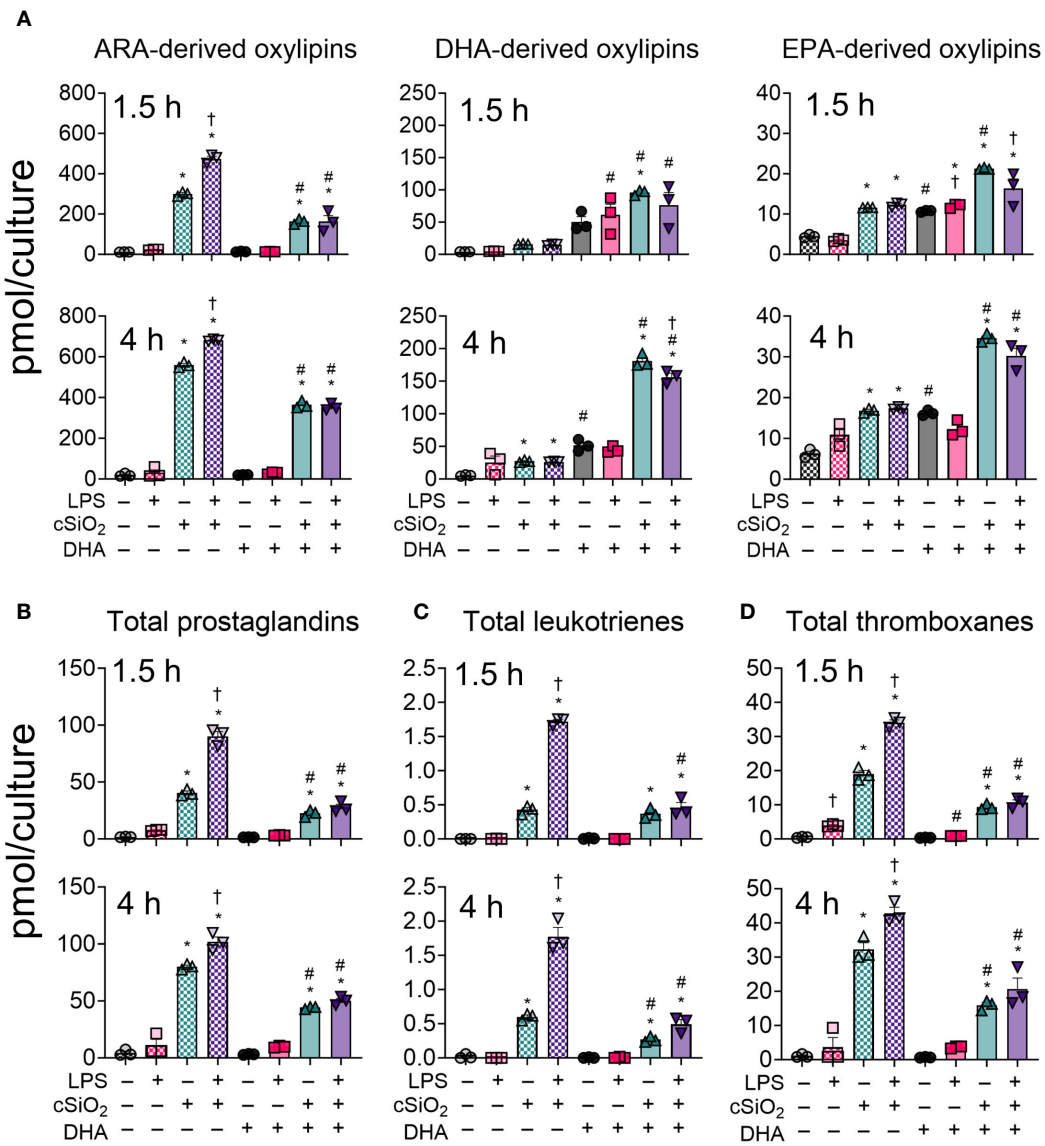
LPS, cSiO_2_, and DHA differentially impact generation of ARA-, DHA-, and EPA-derived oxylipins from FLAMs. FLAMs were treated with ethanolic DHA (25 µM) or ethanol vehicle (VEH) for 24 h, primed with LPS (20 ng/ml), and/or exposed to cSiO_2_ (12.5 µg/cm^2^). **(A)** Total ARA-, DHA-, and EPA-derived oxylipins were quantified for all experimental groups at 1.5 h and 4 h post cSiO_2_. **(B)** Total prostaglandins, **(C)** leukotrienes, and **(D)** thromboxanes were quantified for all experimental groups at 1.5 h and 4 h post cSiO_2_. Data are shown as mean ± SEM. MetaboAnalyst Version 5.0 was used for data normalization and statistically significant differences were determined by one-way analysis of variance (ANOVA) (FDR = 0.05) followed by Tukey’s honestly significant difference (HSD) *post-hoc* test. *FDR q<0.05 for cSiO_2_ vs. controls; ^#^FDR q<0.05 for DHA vs. controls; ^†^FDR q<0.05 for LPS vs. controls.

The effects of cSiO_2_ on the combined intracellular and extracellular lipidome in VEH- and DHA-treated FLAMs was compared in unprimed and LPS-primed FLAMs (**Figure 2A**) and are illustrated in **Figure 2B** and **Supplementary Figure 1**. In addition, summarized oxylipin quantities (**Supplementary Tables 3**-**5**), and individual oxylipin quantities (50) were compared between experimental groups at each designated timepoint. Oxylipin profile shifts were highly pronounced following cSiO_2_ exposure (**Figure 2B**); therefore, we focused our analysis on the 1.5 h and 4 h post cSiO_2_ timepoints. cSiO_2_ elevated total levels of ARA-derived oxylipins and DHA-/EPA-derived oxylipins produced from VEH- and DHA-treated FLAMs, respectively (**Figure 3A**, **Supplementary Tables 4, 5**). In DHA-treated FLAMs, cSiO_2_-induced levels of ARA-derived oxylipins were significantly decreased, and, correspondingly, cSiO_2_-induced levels of DHA-/EPA-derived oxylipins were significantly elevated. cSiO_2_-induced levels of EPA-derived oxylipins also increased modestly in VEH-treated FLAMs. LPS priming elicited a marked increase in cSiO_2_-triggered ARA-derived oxylipins production in VEH-treated FLAMs and a marked decrease in cSiO_2_-triggered EPA-derived oxylipins levels in DHA-treated FLAMs. Findings at 4 h post cSiO_2_ reflected those found at 1.5 h with higher overall quantities of ARA-, EPA-, and DHA-derived metabolites.

### DHA suppresses cSiO_2_-induced production of ARA-derived prostaglandins, leukotrienes, and thromboxanes in unprimed and LPS-primed FLAMs

**Figure 4:**
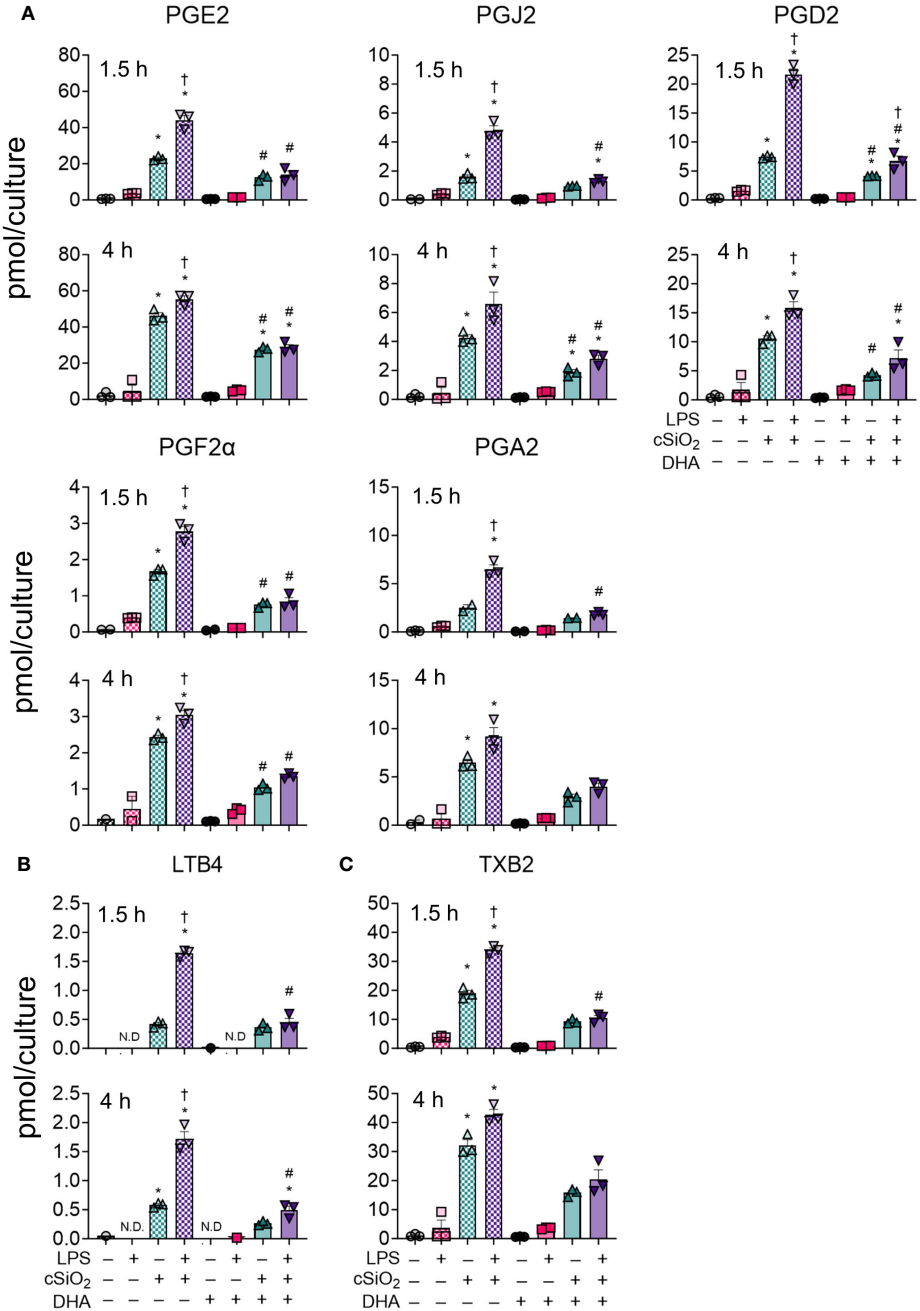
DHA dampens cSiO_2_-induced production of ARA-derived prostaglandins, LTB4, and TXB2 in FLAMs. **(A)** PGE2, PGJ2, PGD2, PGF2α, and PGA2, **(B)** LTB4, and **(C)** TXB2 were quantified by LC-MS for all experimental groups at 1.5 h and 4 h post cSiO_2_. Treatment conditions were tested using three biological replicates, and oxylipins were measured using one technical replicate per sample. Data are shown as mean ± SEM. MetaboAnalyst Version 5.0 was used for data normalization and statistical analysis by one-way analysis of variance (ANOVA) (FDR = 0.05) followed by Tukey’s honestly significant difference (HSD) *post-hoc* test. *FDR q<0.05 for cSiO_2_ vs. controls; ^#^FDR q<0.05 for DHA vs. controls; ^†^FDR q<0.05 for LPS vs. controls.

At 1.5 h and 4 h, cSiO_2_ induced robust increases in total prostaglandins (**Figure 3B**), leukotrienes (**Figure 3C**) and thromboxanes (**Figure 3D**) compared to VEH-treated FLAMs (**Supplementary Tables 4**, **5**). Levels of representative oxylipins from each metabolite class including PGE2, PGJ2, PGD2, PGF2α, and PGA2 (**Figure 4A**), LTB4 (**Figure 4B**), and TXB2 (**Figure 4C**) were increased in like manner in the presence of cSiO_2_ (50).

LPS priming augmented cSiO_2_-triggered production of total and individual prostaglandins, leukotrienes, and thromboxanes (**Figures 3B-D**, **4**). DHA significantly reduced cSiO_2_-induced prostaglandin, leukotriene, and thromboxane production, yet induction of these metabolites was still significant compared to baseline levels in DHA-treated FLAMs. Interestingly, LPS priming did not significantly impact cSiO_2_-induced levels of prostaglandins, leukotrienes, and thromboxanes in DHA-treated FLAMs.

### DHA broadly skews cSiO_2_-induced hydroxy fatty acids in FLAMS from being ω-6 PUFA-derived to being ω-3 PUFA-derived

**Figure 5:**
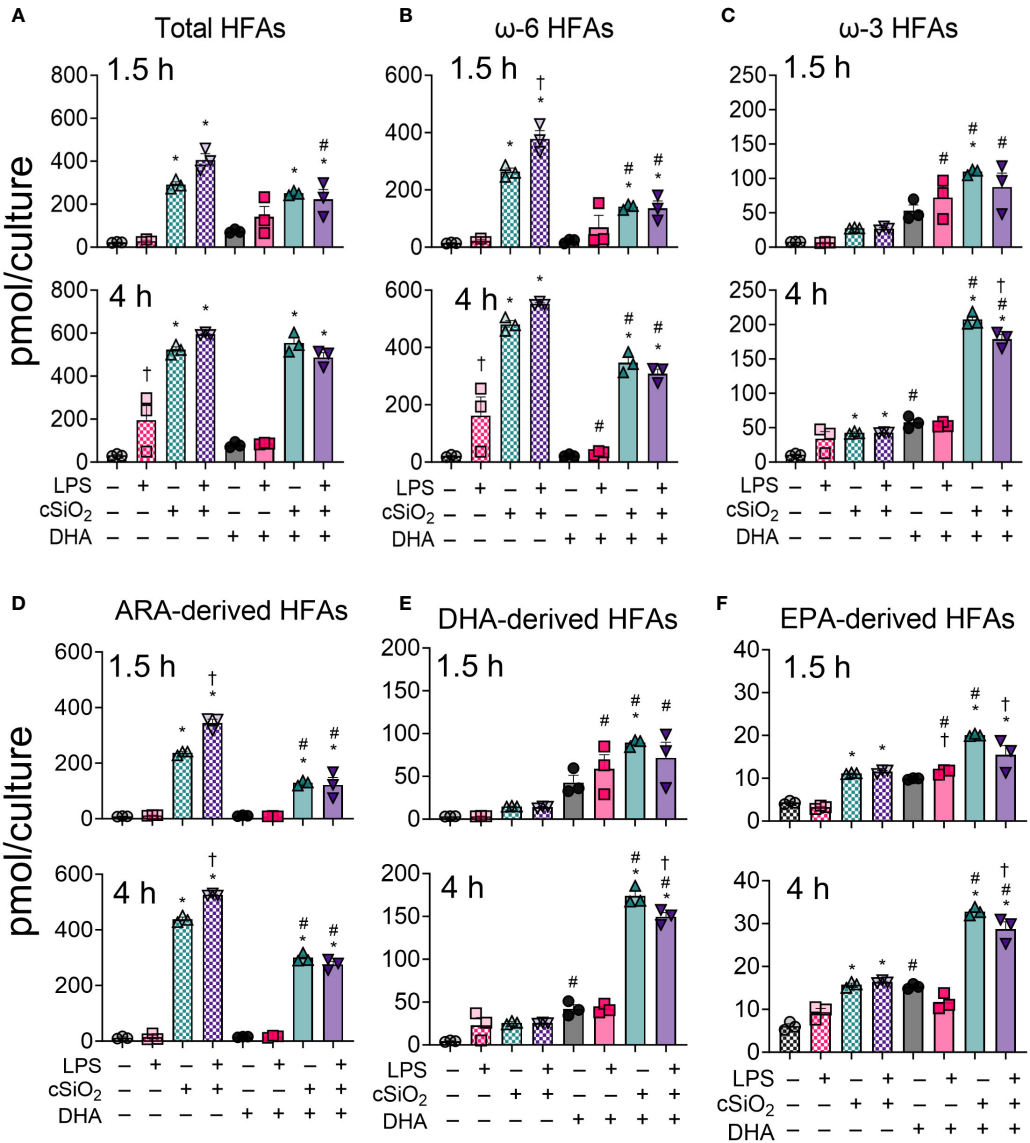
DHA skews cSiO_2_-induced hydroxy fatty acid (HFA) metabolites from being ω-6 PUFA-derived and toward being ω-3 PUFA-derived. **(A)** Total hydroxy fatty acids (HFAs), **(B)** ω-6 HFAs, **(C)** ω-3 HFAs, **(D)** ARA-derived HFAs, **(E)** DHA-derived HFAs, and **(F)** EPA-derived HFAs were quantified by LC-MS for all experimental groups at 1.5 h and 4 h post cSiO_2_. Treatment conditions were tested using three biological replicates, and oxylipins were measured using one technical replicate per sample. Data are shown as mean ± SEM. MetaboAnalyst Version 5.0 was used for data normalization and statistically significant differences were determined by one-way analysis of variance (ANOVA) (FDR = 0.05) followed by Tukey’s honestly significant difference (HSD) *post-hoc* test. *FDR q<0.05 for cSiO_2_ vs. controls; ^#^FDR q<0.05 for DHA vs. controls; ^†^FDR q<0.05 for LPS vs. controls.

Total hydroxy fatty acid (HFA) metabolites were significantly increased by cSiO_2_ exposure in both VEH-treated and DHA-treated FLAMs at 1.5 h and 4 h (**Figure 5A**, **Supplementary Tables 4**, **5**). DHA supplementation significantly reduced ω-6 HFAs and increased ω-3 HFAs at both timepoints (**Figures 5B, C**). The suppressive effect of DHA on ω-6 HFA levels was more marked at 1.5 h than at 4 h, while ω-3 HFA levels steadily increased in DHA-treated FLAMs over time. LPS priming further increased levels of cSiO_2_-induced ω-6 HFAs at 1.5 h and decreased levels of cSiO_2_-induced ω-3 HFAs at 4 h.

Observed changes in ARA-derived HFAs reflected those in total ω-6 HFAs (**Figures 5B, D**, **Supplementary Tables 4**, **5**). cSiO_2_ triggered significant increases in ARA-derived HFA levels in both VEH-treated and DHA-treated FLAMs (**Figure 5D**). DHA significantly reduced metabolite levels, and LPS priming further potentiated cSiO_2_-induced metabolite production at 1.5 h and 4 h. Likewise, DHA- and EPA-derived HFA levels reflected total levels of ω-3 HFAs, as these significantly increased in DHA-treated cells exposed to cSiO_2_ starting at 1.5 h and continuing at 4 h (**Figures 5C, E**, **Supplementary Tables 4**, **5**). In both VEH-treated FLAMs and DHA-treated FLAMs, quantities of ARA-, EPA-, and DHA-derived HFAs were ranked as follows for both timepoints: ARA > DHA > EPA (**Figures 5D-F**). Furthermore, ARA-derived HFAs accounted for the majority of total measured ω-6 HFAs, whereas EPA and DHA both accounted for the majority of total measured ω-3 HFAs.

### DHA suppresses cSiO_2_-induced production of ARA-derived HETEs and induces production of DHA-derived HDoHEs and EPA-derived HEPEs

**Figure 6:**
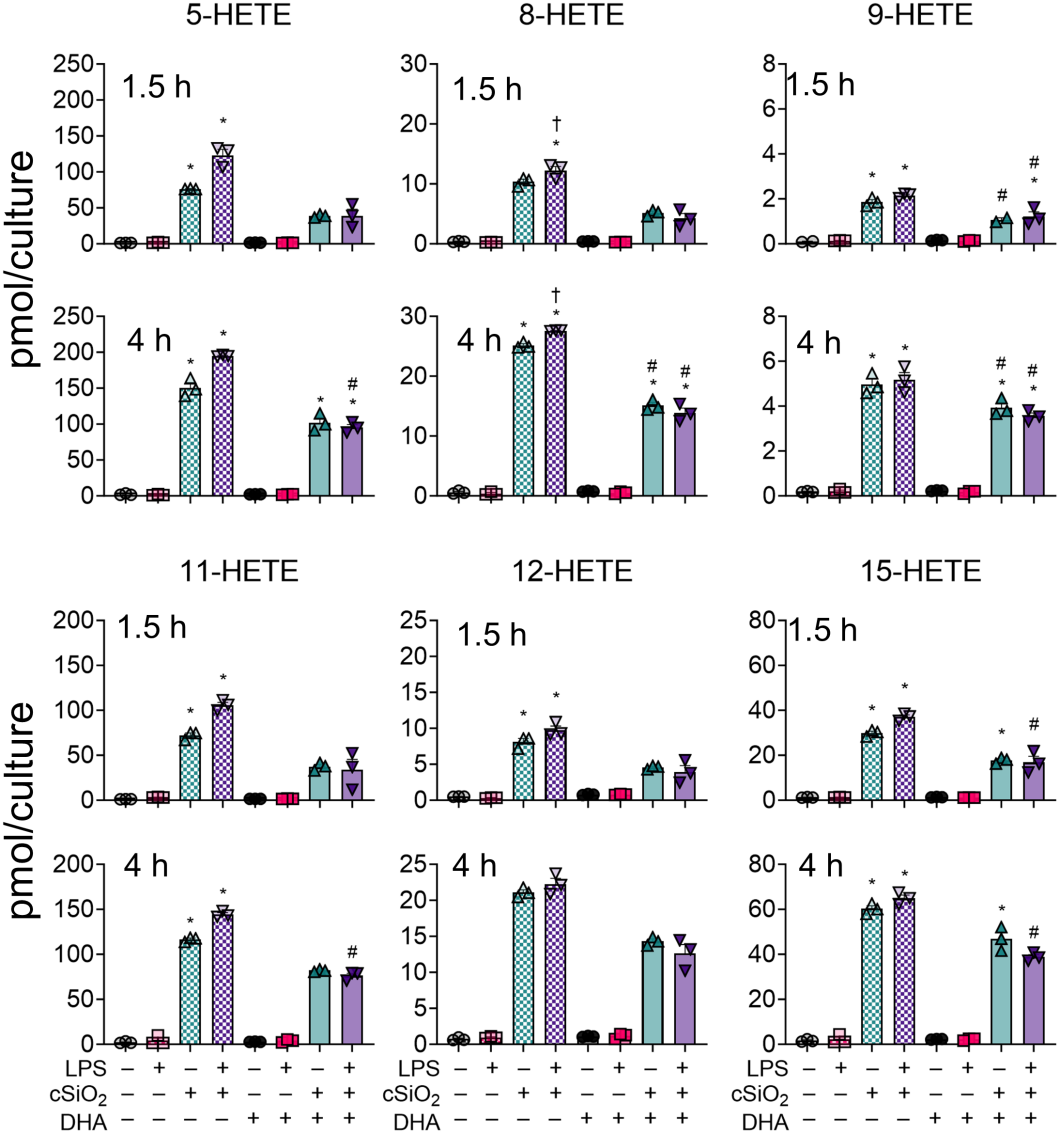
cSiO_2_-induced production of ARA-derived HFAs is diminished with DHA supplementation. 5-HETE, 8-HETE, 9-HETE, 11-HETE, 12-HETE, and 15-HETE were quantified by LC-MS for all experimental groups at 1.5 h and 4 h post cSiO_2_. Treatment conditions were tested using three biological replicates, and oxylipins were measured using one technical replicate per sample. Data are shown as mean ± SEM. MetaboAnalyst Version 5.0 was used for data normalization and statistically significant differences were determined by one-way analysis of variance (ANOVA) (FDR = 0.05) followed by Tukey’s honestly significant difference (HSD) *post-hoc* test. *FDR q<0.05 for cSiO_2_ vs. controls; ^#^FDR q<0.05 for DHA vs. controls; ^†^FDR q<0.05 for LPS vs. controls.

cSiO_2_-induced ω-6 HFAs in VEH-treated FLAMs at 1.5 h and 4 h consisted primarily of ARA-derived 5-HETE, 8-HETE, 9-HETE, 11-HETE, 12-HETE, and 15-HETE (**Figure 6**) (50). Relative abundance of selected HETEs was: 5-HETE > 11-HETE > 15-HETE > 8-HETE > 12-HETE > 9-HETE. HETE levels also increased in DHA-treated FLAMs exposed to cSiO_2_ but were found to be significant only for 5-HETE, 8-HETE, and 9-HETE at 4 h and 15-HETE at both timepoints. In line with total ARA-derived HFA levels (**Figure 5D**), DHA significantly suppressed 8-HETE at 4 h and 9-HETE at both timepoints (**Figure 6**). Levels of other cSiO_2_-induced HETEs (e.g., 5-HETE, 11-HETE, 12-HETE, 15-HETE) also were reduced in DHA-treated FLAMs, but the findings were not statistically significant. LPS priming elicited a significant increase in cSiO_2_-induced 8-HETE and a non-significant trend toward increased levels of other HETEs in VEH-treated FLAMs at both timepoints.

**Figure 7:**
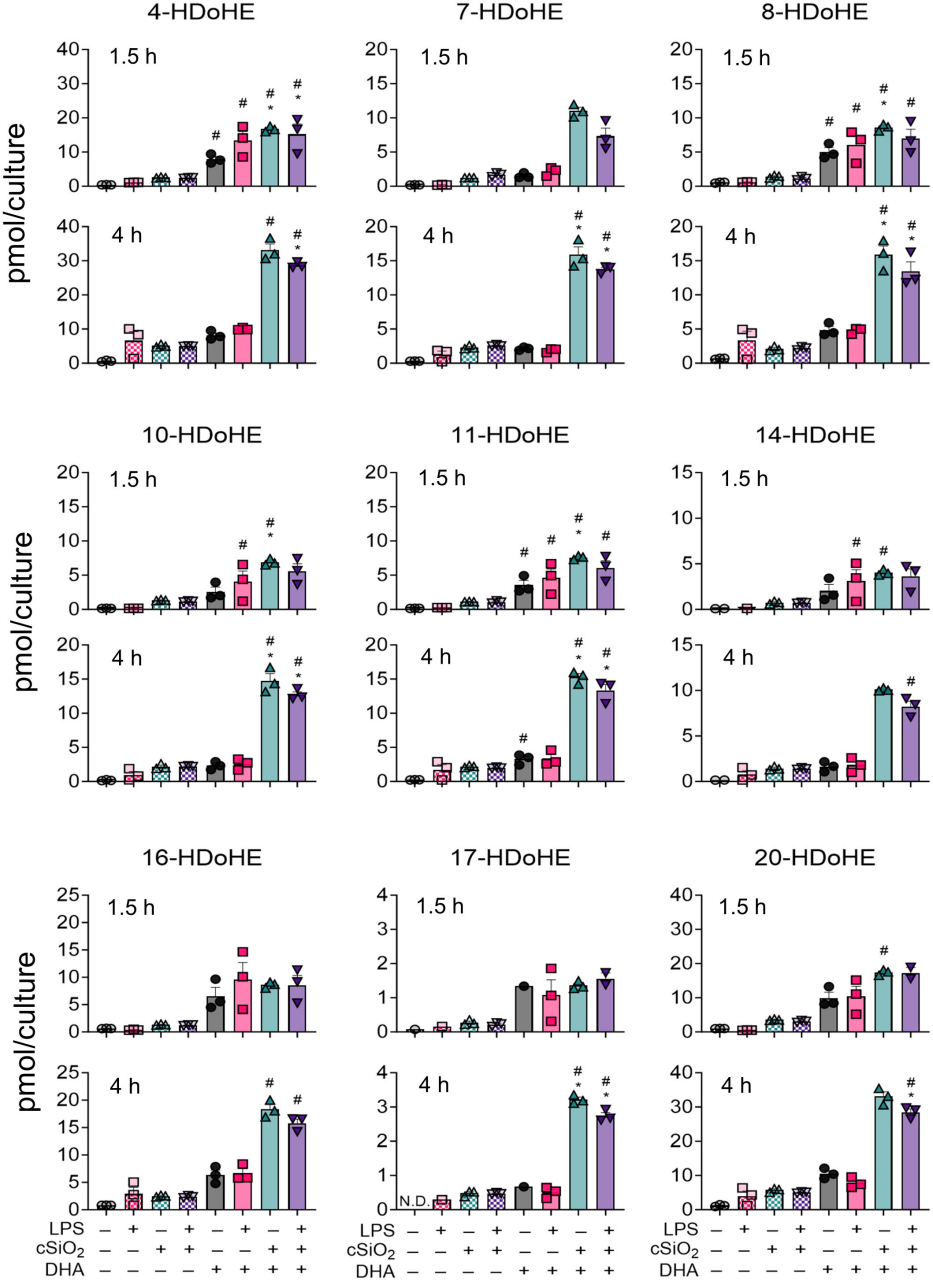
cSiO_2_ exposure of DHA-supplemented FLAMs triggers increased production of DHA-derived hydroxy fatty acids (HFAs). 4-HDoHE, 7-HDoHE, 8-HDoHE, 10-HDoHE, 11-HDoHE, 14-HDoHE, 16-HDoHE, 17-HDoHE, and 20-HDoHE were quantified by LC-MS for all experimental groups at 1.5 h and 4 h post cSiO_2_. Treatment conditions were tested using three biological replicates, and oxylipins were measured using one technical replicate per sample. Data are shown as mean ± SEM. MetaboAnalyst Version 5.0 was used for data normalization and statistically significant differences were determined by one-way analysis of variance (ANOVA) (FDR = 0.05) followed by Tukey’s honestly significant difference (HSD) *post-hoc* test. *FDR q<0.05 for cSiO_2_ vs. controls; ^#^FDR q<0.05 for DHA vs. controls; ^†^FDR q<0.05 for LPS vs. controls.

In DHA-treated FLAMs, cSiO_2_ induced significant increases in several DHA-derived HDoHEs (i.e., 4-HDoHE, 7-HDoHE, 8-HDoHE, 10-HDoHE, 11-HDoHE, 14-HDoHE, 16-HDoHE, 17-HDoHE, 20-HDoHE) (**Figure 7**) and in several EPA-derived HEPEs (i.e., 5-HEPE, 8-HEPE, 9-HEPE, 11-HEPE, 12-HEPE, 15[S]-HEPE) at both timepoints (**Supplementary Figure 2**) (50). Relative abundance of selected HDoHEs was: 4-HDoHE ≈ 20-HDoHE > 16-HDoHE > 7-HDoHE ≈ 8-HDoHE ≈ 10-HDoHE ≈ 11-HDoHE > 14-HDoHE > 17-HDoHE and relative abundance of selected HEPEs was: 5-HEPE > 8-HEPE ≈ 11-HEPE > 15(S)-HEPE > 12-HEPE > 9-HEPE. While cSiO_2_ exposure led to significant increases in EPA-derived 5-HEPE, 11-HEPE, and 12-HEPE in VEH-treated cells during the time-course, DHA-derived HDoHEs did not undergo significant increases in VEH-treated cells exposed to cSiO_2_. The effects of LPS priming on cSiO_2_-induced HEPEs and HDoHEs were minimal at 1.5 h and 4 h.

### DHA modestly influences production of the specialized pro-resolving lipid mediators RvD6 (4,17-DiHDoPE) and MaR1_ω-3 DPA_ in FLAMs

**Figure 8:**
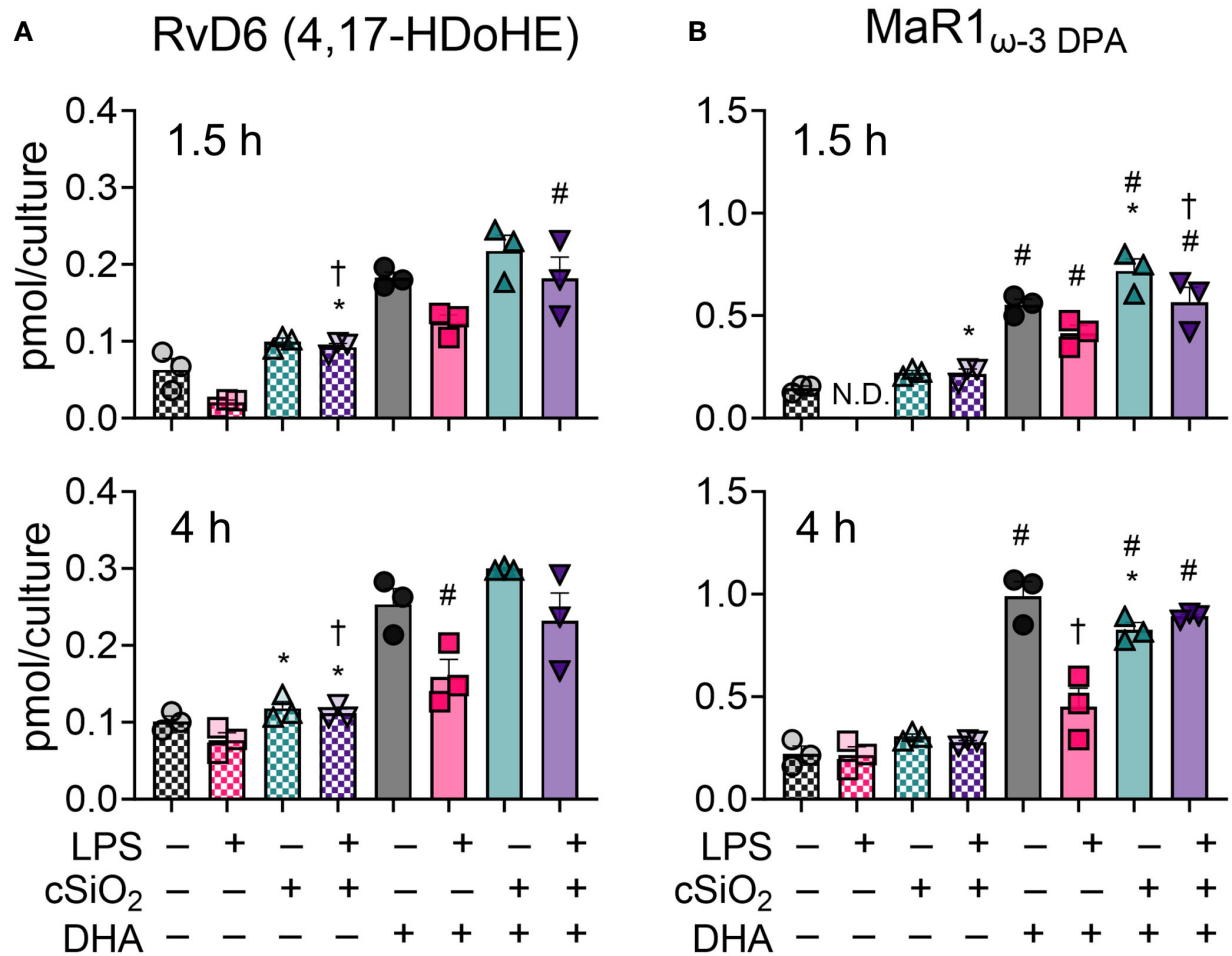
DHA supplementation contributes to modest production of specialized pro-resolving mediators RvD6 (4,17-DiHDoPE) and MaR1_ω-3 DPA_ in FLAMs. **(A)** RvD6 (4,17-DiHDoPE) and **(B)** MaR1_ω-3 DPA_ were quantified by LC-MS for all experimental groups at 1.5 h and 4 h post cSiO_2_. Treatment conditions were tested using three biological replicates, and oxylipins were measured using one technical replicate per sample. Data are shown as mean ± SEM. MetaboAnalyst Version 5.0 was used for data normalization and statistical analysis by one-way analysis of variance (ANOVA) (FDR = 0.05) followed by Tukey’s honestly significant difference (HSD) *post-hoc* test. *FDR q<0.05 for cSiO_2_ vs. controls; ^#^FDR q<0.05 for DHA vs. controls; ^†^FDR q<0.05 for LPS vs. controls.

Specialized pro-resolving mediators (SPMs) are a class of oxylipins comprised of resolvins, maresins, protectins, and lipoxins derived from ARA, EPA, ω-3 DPA, and DHA that limit proinflammatory cytokine release and promote dead cell clearance by macrophages (56–58). Most SPMs assessed in our LC-MS oxylipin panel were not detected at any timepoint (50). DHA supplementation did, however, cause a modest increase in RvD6 (4,17-DiHDoPE) and MaR1_ω-3 DPA_ at 1.5 h and 4 h (**Figure 8**) (50). In DHA-treated FLAMs, cSiO_2_ exposure did not significantly influence production of RvD6 at either timepoint (**Figure 8A**) but significantly increased MaR1_ω-3 DPA_ at 1.5 h and decreased MaR1_ω-3 DPA_ at 4 h (**Figure 8B**). LPS priming significantly suppressed MaR1_ω-3 DPA_ production in DHA-treated FLAMs at 4 h, modestly inhibited MaR1_ω-3 DPA_ at 1.5 h, and modestly decreased RvD6 production at both 1.5 h and 4 h.

### DHA modestly influences production of EpFAs and DiHFAs in cSiO_2_-exposed FLAMs

**Figure 9:**
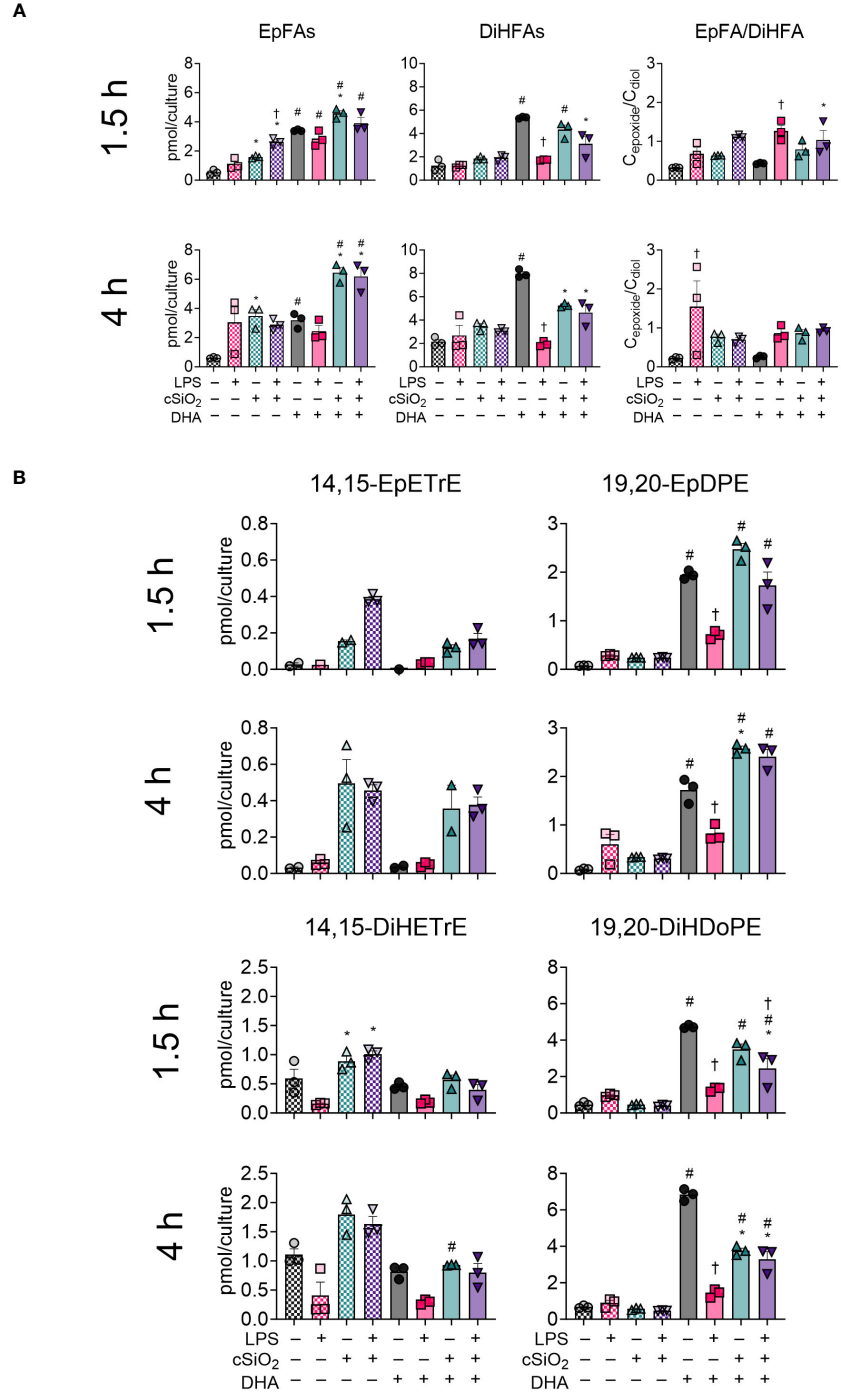
cSiO_2_ exposure and DHA supplementation contribute to increased production of epoxy fatty acids (EpFAs) and dihydroxy fatty acids (DiHFAs). **(A)** Total EpFAs, total DiHFAs, and EpFA : DiHFA ratios (C_epoxide_/C_diol_) were quantified by LC-MS for all experimental groups at 1.5 h and 4 h post cSiO_2_. **(B)** 14,15-EpETrE, 19,20-EpDPE, 14,15-DiHETrE, and 19,20-DiHDoPE were quantified for all experimental groups at 1.5 h and 4 h post cSiO_2_. Treatment conditions were tested using three biological replicates, and oxylipins were measured using one technical replicate per sample. Data are shown as mean ± SEM. MetaboAnalyst Version 5.0 was used for data normalization and statistical analysis by one-way analysis of variance (ANOVA) (FDR = 0.05) followed by Tukey’s honestly significant difference (HSD) *post-hoc* test. *FDR q<0.05 for cSiO_2_ vs. controls; ^#^FDR q<0.05 for DHA vs. controls; ^†^FDR q<0.05 for LPS vs. controls.

Total epoxy fatty acids (EpFAs) and CYP450-derived dihydroxy fatty acids (DiHFAs) were quantified from VEH-treated and DHA-treated FLAMs (**Figure 9A**, **Supplementary Tables 4**, **5**). In VEH-treated FLAMs, cSiO_2_ modestly induced production of EpFA metabolites at 1.5 h and 4 h and did not significantly impact production of DiHFA metabolites. Conversely, cSiO_2_ triggered significant increases in total EpFAs and DiHFAs in DHA-treated FLAMs at both timepoints. Interestingly, LPS priming significantly reduced total DiHFA metabolite levels in the absence of cSiO_2_ in DHA-treated FLAMs. Separate and simultaneous LPS priming and cSiO_2_ exposure elicited modest increases in EpFA : DiHFA ratios in both VEH-treated and DHA-treated FLAMs during the time-course.

Effects of cSiO_2_ and DHA were also analyzed for selected CYP450 oxylipin products of ARA (i.e., 14,15-EpETrE, 14,15-DiHETrE) and DHA (i.e., 19,20-EpDPE, 19,20-DiHDoPE) (**Figure 9B**) (50). cSiO_2_ evoked production of 14,15-EpETrE and 14,15-DiHETrE starting at 1.5 h and continuing through 4 h in VEH-treated FLAMs and, to a lesser degree, in DHA-treated FLAMs. LPS priming also modestly increased cSiO_2_-triggered production of 14,15-EpETrE in VEH-treated FLAMs. Changes in 14,15-EpETrE levels were not significant, and cSiO_2_-induced production of 14,15-DiHETrE was significant only at 1.5 h. In contrast, DHA treatment promoted robust production of 19,20-EpDPE and 19,20-DiHDoPE at both timepoints. Exposure to cSiO_2_ resulted in a subtle, yet non-significant, increase in 19,20-EpDPE and corresponding decrease in 19,20-DiHDoPE at both timepoints. Intriguingly, LPS priming alone significantly decreased levels of 19,20-EpDPE and 19,20-DiHDoPE during the experiment. Overall, levels of 19,20-EpDPE and 19,20-DiHDoPE were found to be higher than levels of 14,15-EpETrE and 14,15-DiHETrE.

### DHA suppresses cSiO_2_-induced release of proinflammatory cytokines from LPS-primed FLAMs

**Figure 10:**
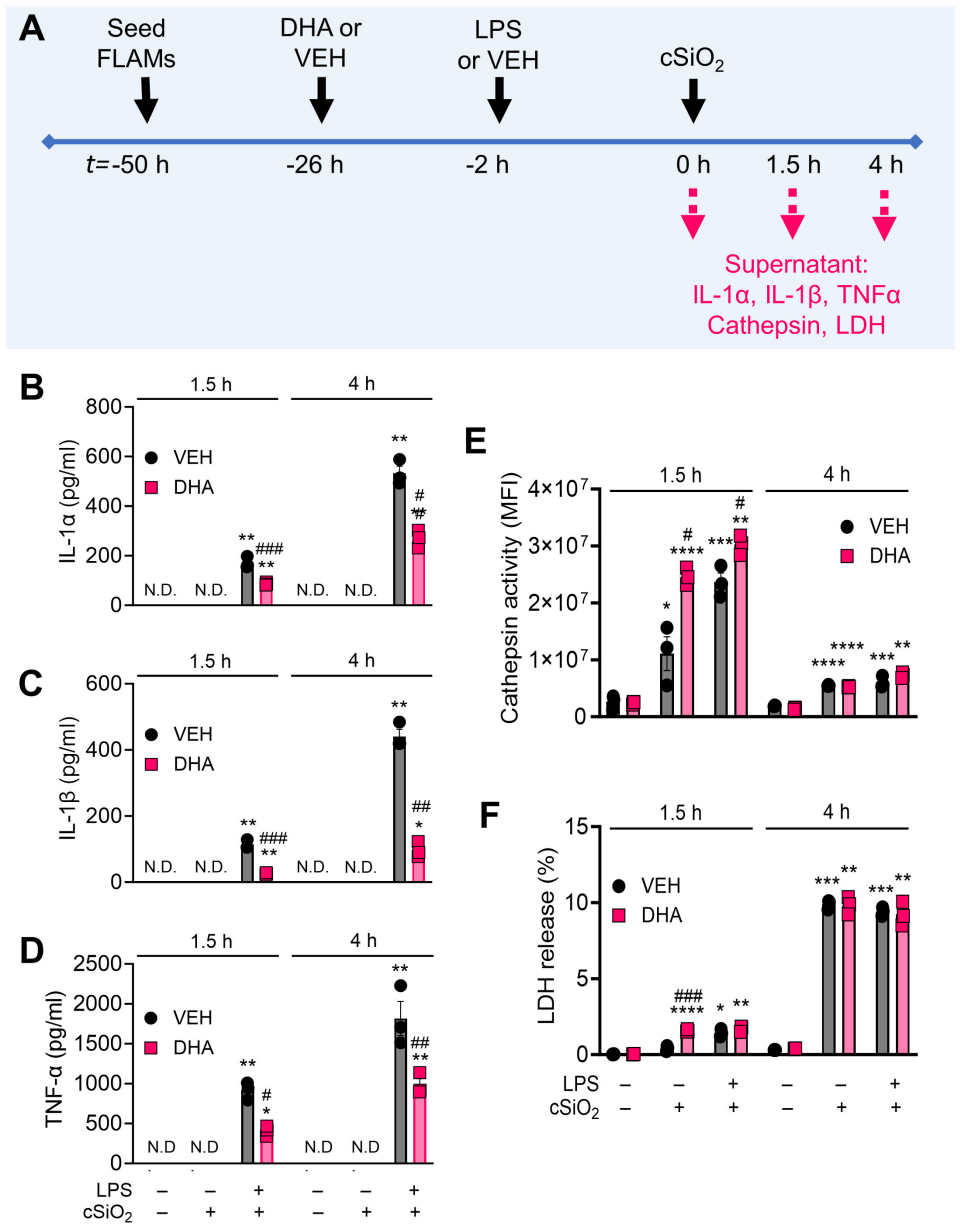
DHA suppresses cSiO_2_-induced release of proinflammatory cytokines in LPS-stimulated FLAMs. **(A)** FLAMs were treated with ethanolic DHA (25 µM) or ethanol vehicle (VEH) for 24 h, primed with LPS (20 ng/ml), then exposed to cSiO_2_ (12.5 µg/cm^2^). Cell culture supernatants were collected at t = 1.5 h and 4 h post cSiO_2_, and **(B)** IL-1α, **(C)** IL-1β, and **(D)** TNF-α were quantified by ELISA. **(E)** Lysosomal cathepsin activity (expressed in units of mean fluorescence intensity [MFI]) was quantified as a metric for lysosomal membrane permeabilization, and **(F)** percent LDH release was quantified as a metric for cell death. *p<0.05, **p<0.01, ***p<0.001, ****p<0.0001: Statistically significant differences between cSiO_2_ and its corresponding control. ^#^p<0.05, ^##^p<0.01, ^###^p<0.001: Statistically significant differences between DHA and its corresponding control. N.D., not determined.

The concurrent impacts of DHA on proinflammatory cytokine release from unprimed and LPS-primed FLAMs were evaluated at 1.5 h and 4 h post cSiO_2_-treatment (**Figure 10A**). At both timepoints, unprimed FLAMs treated with either VEH or cSiO_2_ alone secreted negligible amounts of IL-1α, IL-1β, and TNF-α (**Figures 10B–D**). In contrast, LPS-primed FLAMs released robust amounts of IL-1α, IL-1β, and TNF-α at both timepoints following cSiO_2_ exposure, with much higher cytokine levels observed at 4 h compared to 1.5 h post cSiO_2_. DHA pretreatment in LPS-primed FLAMs markedly reduced cSiO_2_-induced release of IL-1α, IL-1β, and TNF-α release at both timepoints. No notable DHA effects on proinflammatory cytokine release were evident in FLAMs treated with VEH or cSiO_2_ alone.

### DHA does not suppress cSiO_2_-induced cathepsin or LDH release in FLAMs

In addition to analyzing the impacts of DHA, LPS, and cSiO_2_ on the cellular lipidome, we also measured lysosomal cathepsin and LDH activities in collected supernatants (**Figure 10A**). Cathepsin activity in VEH-treated cells exposed to cSiO_2_ alone was higher at 1.5 h (1.1×10^7^ MFI) than at 4 h (5.5×10^6^ MFI) post cSiO_2_ treatment (**Figure 10E**). Interestingly, DHA caused a moderate increase in cathepsin activity in FLAMs exposed to cSiO_2_ alone or to both LPS and cSiO_2_ at 1.5 h but not at 4 h. LDH release in VEH-treated FLAMs treated with cSiO_2_ alone was higher at 4 h (9.9%) than at 1.5 h (0.4%) (**Figure 10F**). LPS priming of FLAMs increased cSiO_2_ -induced extracellular lysosomal cathepsin and LDH response at 1.5 h post cSiO_2_ in both VEH- and DHA-treated FLAMs but not at 4 h post cSiO_2_ (**Figures 10E, F**). In line with cathepsin activity analyses, DHA caused a slight increase in LDH release in FLAMs exposed to cSiO_2_ alone at 1.5 h and did not significantly impact LDH release at 4 h.

### DHA does not influence cSiO_2_-induced lysosomal membrane permeabilization, mitochondrial toxicity, or death in FLAMs

**Figure 11:**
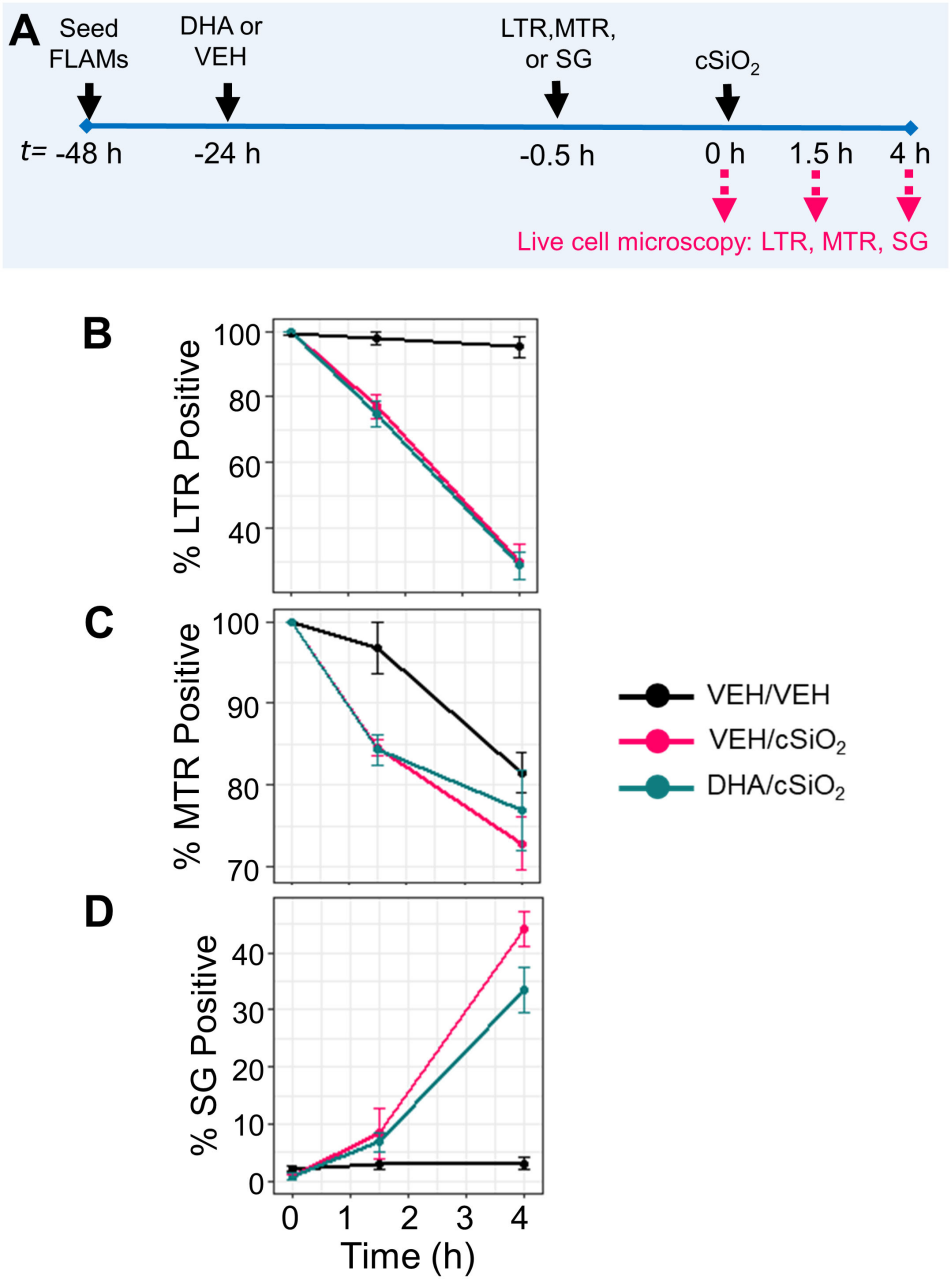
DHA does not affect early cSiO_2_-induced lysosomal membrane permeabilization, mitochondrial toxicity, and death in FLAMs. **(A)** FLAMs were treated with ethanolic DHA (25 µM) or ethanol vehicle (VEH) for 24 h. VEH-treated and DHA-treated FLAMs were then stained with LysoTracker Red (LTR; 50 nM), MitoTracker Red (MTR; 25 nM), or SYTOX Green (SG; 200 nM) in DPBS^+/+^ for 30 min. After 30 minutes to allow fluorescent dyes to equilibrate, cSiO_2_ was added dropwise at 0 or 12.5 μg/cm^2^. **(B)** Percent LTR^+^, **(C)** MTR^+^, and **(D)** SG^+^ cells from 0 to 4 h post cSiO_2_ in VEH/VEH, VEH/cSiO_2_, and DHA/cSiO_2_ treated cells quantified using CellProfiler 4.2.1 and RStudio Desktop. Data are shown as mean ± SEM.

Live-cell imaging using LysoTracker Red (LTR), MitoTracker Red (MTR), and SYTOX Green (SG) was employed to further assess the impacts of DHA on cSiO_2_-induced lysosomal membrane permeabilization (LMP), mitochondrial depolarization, and cell death, respectively (**Figure 11A**, **Supplementary Figure 3A**). In a preliminary experiment, we found that LPS priming slightly expedited cSiO_2_-induced loss of LTR^+^ cells (**Supplementary Figure 3B**). LPS did not significantly impact cSiO_2_-induced development of SG^+^ cells (**Supplementary Figure 3D**). Intriguingly, LPS priming perpetuated cSiO_2_-triggered loss of MTR^+^ cells (**Supplementary Figure 3C**). In unprimed FLAMs, the proportions of LTR^+^ cells (**Figure 10B**), MTR^+^ cells (**Figure 11C**), and SG^+^ cells (**Figure 11D**) at time of cSiO_2_ addition were nearly 100%, 100%, and 0%, respectively, relative to total cells. Loss of lysosomal integrity occurred at very similar rates up to 4 h post cSiO_2_ in VEH- and DHA-treated unprimed FLAMs exposed to cSiO_2_ (**Figure 11B**). Mitochondrial depolarization progressed at approximately the same rate in VEH- and DHA-treated unprimed FLAMs from 0 to 1.5 h, and DHA slightly protected FLAMs from further mitochondrial depolarization from 1.5 to 4 h (**Figure 11C**). Minimal cell death was observed from 0 to 1.5 h for both VEH- and DHA-treated cells, and DHA slightly, albeit insignificantly, suppressed cell death from 1.5 to 4 h (**Figure 11D**). Interestingly, DHA pretreatment modestly enhanced cSiO_2_-induced loss of LTR^+^ (**Figure 11B**) and MTR^+^ cells (**Figure 10C**) while increasing SG^+^ cells (**Figure 11D**). Altogether, DHA had negligible effects on cSiO_2_-induced lysosomal membrane permeabilization, mitochondrial toxicity, and death in FLAMs.

## Discussion

AMs comprise the first line of defense against cSiO_2_ and other inhaled particles (59). Preclinical studies have demonstrated that cSiO_2_ elicits robust proinflammatory mediators and death in AMs, which might be critical first steps for subsequent development of inflammation and autoimmunity observed in the lungs of animals exposed to the particle. Importantly, intervention by dietary DHA supplementation dramatically suppresses these responses (27, 60, 61). While bioactive oxylipins potentially play key roles in these responses, the effects of cSiO_2_ and DHA on oxylipin production in naïve and TLR-activated AMs have not yet been systematically addressed. Herein, we hypothesized that DHA would inhibit cSiO_2_-induced production of proinflammatory eicosanoids in FLAMs. This investigation is the first to comprehensively assess how LPS, cSiO_2_, and DHA time-dependently influence the oxylipin signature in an AM surrogate. Several novel findings were made. First, DHA delivery to FLAM cultures as either an ethanolic suspension or as BSA complexes were equally effective in displacing the ω-6 PUFA ARA and ω-9 PUFA OA from cellular phospholipids, resulting in increased percent ω-3 PUFAs and ω-3 HUFA scores. Second, cSiO_2_ exposure within 4 h elicited an ARA-derived eicosanoid storm consisting of prostaglandins, leukotrienes, thromboxanes, and HETEs coupled with less prominent changes in ω-6 EpFAs and DiHFAs. Third, supplementing FLAMs with DHA dramatically suppressed cSiO_2_-induced ARA-derived oxylipins while promoting production of DHA- and EPA-derived oxylipins, including HDoHEs and HEPEs. Fourth, LPS priming modestly enhanced cSiO_2_-induced ARA-derived oxylipin generation. Finally, within a concurrent time period, DHA suppressed cSiO_2_-triggered release of IL-1α, IL-1β, and TNF-α in LPS-primed FLAMs but modestly enhanced both cSiO_2_-induced decrements in lysosome and mitochondrial integrity concurrently and cell death.

Preclinical and clinical studies suggest that increased ω-3 PUFA intake—and consequently increased ω-3 PUFA tissue content—are associated with decreased symptom severity in chronic inflammatory conditions such as rheumatoid arthritis (62, 63), lupus (23, 64), and cardiovascular disease (65, 66). The pro-resolving impacts of the ω-3 PUFA DHA are multifaceted. At the cellular level, DHA 1) modulates membrane fluidity by displacing ω-6 PUFAs from the sn-2 position of membrane phospholipids, 2) suppresses expression and release of proinflammatory cytokines, 3) competes with ω-6 PUFAs as substrates for fatty acid metabolizing enzymes, and 4) undergoes conversion into several classes of highly pro-resolving oxylipins [reviewed in (25, 67, 68)]. In previously published studies, we have found in several macrophage models that DHA is readily incorporated into membrane phospholipids at the expense of ω-6 ARA and ω-9 OA, suppresses LPS-induced transcription and translation of proinflammatory genes, dampens cSiO_2_-induced proinflammatory cytokine release, and stimulates efferocytosis of cSiO_2_-killed cell corpses (27, 28, 45). Our prior investigations also suggest that DHA protects primary AMs, fetal liver-derived macrophages maintained with GM-CSF without TGF-β, and murine RAW264.7 macrophage-like cells from cSiO_2_-induced death (28), dissimilar to the present study.

Our findings that supplementation of FLAMs with DHA in the form of either an ethanolic suspension or BSA complexes were equivalent correspond with previous findings of Wiesenfeld and coworkers (54), who reported that delivery of DHA as ethanolic suspensions and BSA complexes resulted in roughly equal displacement of ARA by DHA in two different transformed macrophage cell lines. Here, we used a physiologically relevant dose of DHA that resulted in plasma membrane incorporation at levels comparable to erythrocyte DHA content observed in previous *in vivo* studies, where mice were fed a realistic human equivalent dose of 5 g/day (61, 69, 70). However, the cell culture conditions used here do not completely reflect other dietary components that could influence AM inflammatory responses from a translational perspective. For instance, the recommended ω-6/ω-3 ratio is 2-3:1 yet the ω-6/ω-3 ratio in the standard Western diet is approximately 20:1 (71), which may increase the risk of inflammatory ARA-derived oxylipin cascades (72). It would therefore be informative in future lipidomics investigations to treat FLAMs with various ratios of ω-6 PUFAs (e.g., LA, ARA) and ω-3 PUFAs (e.g., EPA, DHA) prior to cSiO_2_ exposure to more closely model dietary patterns in rodent and human studies.

Although our lipid metabolite panel may not account for all potential oxylipin species present in FLAMs, we found here that cSiO_2_ induced production of numerous bioactive oxylipins derived from ARA (e.g., PGE2, LTB4, TXB2, HETEs), DHA (e.g., HDoHEs), and EPA (e.g., HEPEs) in VEH-treated and DHA-treated FLAMs. Oxylipins derived from other less abundant ω-6 PUFAs (e.g., LA, DGLA) were also detected in our analysis, which may play roles in modulating cSiO_2_-triggered toxicity in FLAMs (73). These results suggest that cSiO_2_ may induce PLA2-mediated release of ω-6 PUFAs and ω-3 PUFAs from the sn-2 position of phospholipids in VEH-treated FLAMs and DHA-treated FLAMs, respectively (74), freeing these PUFAs for subsequent conversion into oxylipins inside the cell. A previously published study by Sager and coworkers (75) suggests that cSiO_2_ can induce expression of various PLA2 isozymes in the rat lungs, but the impacts of cSiO_2_ on PLA2 expression and activity remain unresearched at large. Additional studies in FLAMs involving genetic deletion or pharmacological inhibition of different PLA2 isozymes would help to clarify the role of PLA2 in cSiO_2_-induced oxylipin production.

LPS priming enhanced cSiO_2_-induced production of ARA-derived PGE2, LTB4, and TXB2 at both timepoints. Accordingly, our observations suggest that LPS priming may upregulate expression of COX and LOX in FLAMs, contributing to heightened production of classical proinflammatory eicosanoids after cSiO_2_ exposure. Previous studies have shown that LPS-triggered TLR4 activation in macrophages contributes to upregulation and activation of COX and LOX enzymes (76–80). In the context of inflammation and infection, classical eicosanoids are important modulators of the immune response. For instance, LTB4 in neutrophils promotes chemotaxis, enhances phagocytosis, triggers degranulation, and induces ROS production as antimicrobial defenses (81). Contrastingly, the immunoregulatory roles of PGE2 are more nuanced. While PGE2 has been previously shown to upregulate expression of proinflammatory cytokines (e.g., IL-1β, IL-6, IL-23) (82), PGE2 has also been shown to induce production of anti-inflammatory cytokines (e.g., IL-10, TGF-β) (83), suppress LPS-induced TNF-α production (84), inhibit bacterial phagocytosis (85), and stimulate M2-associated gene expression via transcription factor CREB (e.g., *Arg1*, *Mrc1*, *Fizz1*, *Ym1*) in macrophages (83, 86). Based on our study, LPS priming contributed to increased rates of lysosomal and mitochondrial integrity loss, but it is unclear whether augmented eicosanoid biosynthesis directly contributed to these observations, as proinflammatory cytokine production significantly increased after LPS priming.

In addition to promoting biosynthesis of classical proinflammatory eicosanoids, LPS priming also elicited release of the proinflammatory cytokines IL-1α, IL-1β, and TNF-α. It remains unclear whether LPS-stimulated proinflammatory cytokines (e.g., IL-1α, IL-1β, TNF-α) interact with their corresponding receptors (e.g., IL-1R, TNFR1) on neighboring FLAMs to stimulate production of ARA-derived oxylipins. Previous investigations suggest that certain proinflammatory cytokines, including IL-1β and TNF-α, can induce production of PGE2 and TXB2 in various contexts (87–89). In the future, it will be informative to either genetically knock out or pharmacologically inhibit proinflammatory cytokine receptors of interest to clarify the roles that cytokine-receptor signaling might play in influencing the cellular lipidome.

While numerous HFAs can be produced via the LOX or CYP450 enzymatic pathways (29, 90), HFAs can also be produced via non-enzymatic oxidation by reactive oxygen species (ROS) (91–93). Here, HFA levels were not significantly changed after LPS priming, which suggests these oxylipins may result from non-enzymatic conversion triggered by cSiO_2_ instead of conversion by LOX enzymes upregulated by LPS. Relatedly, cSiO_2_ caused steady declines in lysosomal and mitochondrial integrity that occurred at similar rates in VEH-treated and DHA-treated FLAMs and corresponded with increased HFA production. cSiO_2_ uptake by macrophages has been previously demonstrated to increase ROS levels in the cytoplasm and phagolysosome, resulting in LMP (94). Furthermore, mitochondrial depolarization has been shown to occur after cSiO_2_-induced LMP (9), and cSiO_2_ exposure has been linked to increased lysosomal, mitochondrial, and total cytosolic ROS production (95, 96). Although we did not directly measure ROS production in the present study, it is plausible that cSiO_2_-triggered HFA production in FLAMs is largely caused by non-enzymatic oxidation via ROS released from damaged lysosomes and mitochondria, as no subsets of HFAs were selectively produced in our oxylipin panel. Follow-up studies should aim to quantify total ROS and mitochondrial ROS produced from cSiO_2_-exposed FLAMs and utilize antioxidant agents (e.g., *N*-acetylcyteine, Trolox) to elucidate the impacts of ROS on HFA and total oxylipin production.

Interestingly, we found that DHA-treated FLAMs produced minimal SPMs, including RvD6 and MaR1_ω-3 DPA_, in response to LPS and cSiO_2_, which was unexpected because macrophages have been reported to biosynthesize a variety of SPMs in response to infectious agents and other inflammatory stimuli (97–99). We speculate that this occurred because the acute timepoints we selected may not adequately capture the kinetics of SPM biosynthesis. For instance, Dalli and coworkers demonstrated in zymosan-treated mice that MaR1_ω-3 DPA_ was negligible at 4 h post-treatment but accumulated between 12-24 h post-treatment (100). In line with this conjecture, we also speculate that most of the administrated DHA was non-enzymatically converted to HDoHE metabolites following cSiO_2_ exposure, leaving insufficient quantities available for enzymatic conversion into various SPMs. It will therefore be important to extend the time-course in future experiments to better understand the kinetics of SPM production in our FLAM model.

We chose to focus our investigation on C57BL/6-derived FLAMs first because we recently characterized this model from a functional perspective and found that these cells are amenable to genetic modulation. This prompted us to assess whether this model was also amenable to lipidome modulation. Previously, we have demonstrated in female autoimmune-prone NZBWF1 mice that dietary DHA administered at human caloric equivalents of 2 or 5 g/d dose-dependently reduces perivascular leukocyte infiltration and expression of proinflammatory proteins in the lung (61, 70, 101). These changes correspond with increased levels of ω-3 PUFAs in erythrocytes and lungs; suppressed levels of cSiO_2_-induced inflammatory proteins and autoantibodies in bronchoalveolar lavage fluid (BALF) and plasma; and delayed onset of resultant glomerulonephritis and proteinuria. Our use of FLAMs from non-autoimmune C57BL/6 mice somewhat limits the translatability of the present study to other studies analyzing respirable cSiO_2_ as an autoimmune trigger in genetically-susceptible mice and humans. Nevertheless, developing a baseline oxylipin profile for C57BL/6 FLAMs will aid us in future investigations comparing effects of LPS, cSiO_2_, and DHA on the lipidome of FLAMs derived from autoimmune-prone mice (e.g., female NZBWF1 mice). Additional perspectives are needed to understand not only the influence of cSiO_2_ and DHA on the oxylipin signatures of non-autoimmune FLAMs and autoimmune-prone FLAMs but also on the lipidome of primary AMs and whole lung homogenates from non-autoimmune mice and autoimmune-prone mice.

Prior studies of protective effects of DHA against lysosomal toxicity, mitochondrial toxicity, and cell death might be highly dependent on cellular phenotype (27, 28, 102–107). As seen here, DHA did not inhibit cSiO_2_-induced LMP, mitochondrial toxicity, or cell death in FLAMs. DHA’s inability to protect against or even enhance cSiO_2_-triggered cell death in FLAMs suggests that these processes might not be critical targets in DHA-mediated lung protection. Nevertheless, cathepsin release and cell death may be vehicles by which cSiO_2_ drives production of pro-resolving DHA-derived oxylipins in FLAMs, as DHA-derived HDoHE levels rose at similar rates to ARA-derived HETE levels following cSiO_2_ exposure.

One limitation of our investigation is that intracellular and extracellular oxylipin content was pooled for all LC-MS analyses, making it impossible to discern quantities of secreted oxylipins from quantities of non-secreted oxylipins. By conducting LC-MS on separated cell cultures and supernatants, we would be able to better understand not only how cSiO_2_ impacts overall oxylipin production but also how cSiO_2_ impacts individual oxylipin release from FLAMs. Accordingly, prostaglandins, thromboxanes, leukotrienes, HFAs, and other subclasses of oxylipins elicit biological activity through transmembrane G protein-coupled receptors (GPCRs) and intracellular receptors such as PPARγ (108–112). While receptor-mediated biological effects have been reported—some proinflammatory and some pro-resolving—for numerous individual oxylipins (**Supplementary Table 6**), it is very likely that oxylipins elicit their biological activity as mixtures. To this end, it would be of interest to generate conditioned medium containing ARA-derived oxylipins and DHA-derived oxylipins from cSiO_2_-exposed VEH-treated FLAMs and cSiO_2_-exposed DHA-treated FLAMs, respectively, and then measure paracrine effects of the oxylipin mixtures on cSiO_2_-induced toxic responses in separate FLAM cultures.

FLAMs are not only useful for conducting broad genomic and lipidomic screens, but they may also be a useful AM surrogate for screening novel drug candidates *in vitro*. Accordingly, Hoffman and coworkers assessed the toxicity and efficacy of 13 known inhalable compounds using rat NR8383 and human U937-derived AM cell lines (113). This approach could easily be applied to our FLAM model to efficiently screen many drug candidates at once without needing to sacrifice large numbers of mice for primary AM isolation. Toxicity and efficacy results from preliminary drug screens in FLAMs could then be used to inform decisions regarding whether to discontinue or continue development of specific drugs in primary AMs, animal models, and ultimately human beings.

## Conclusions

**Figure 12:**
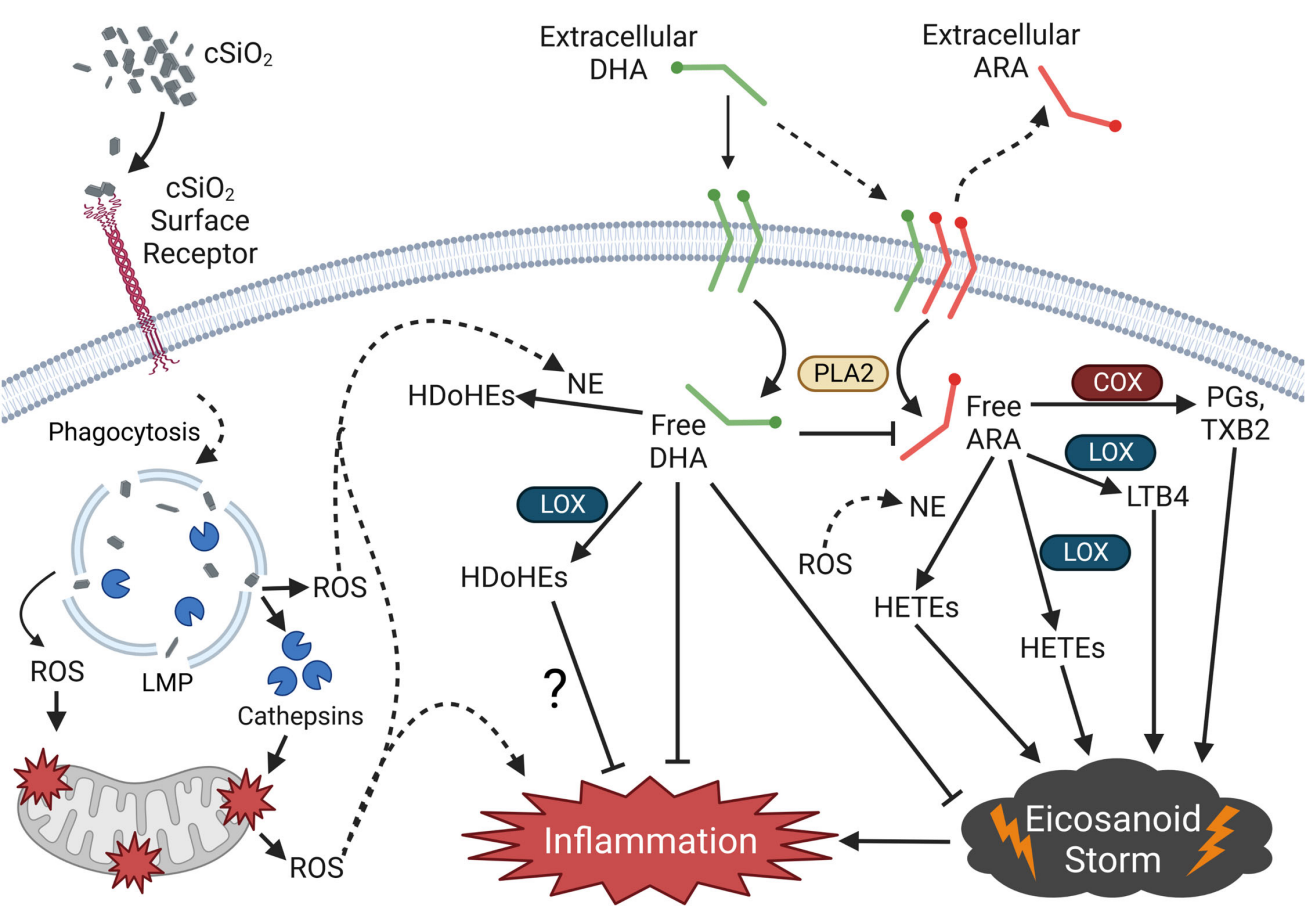
Putative model for the effects of DHA on the cSiO_2_-triggered proinflammatory eicosanoid storm in the FLAM. cSiO_2_ binds a cSiO_2_-specific surface receptor and is phagocytosed by the FLAM. Following phagocytosis, cSiO_2_ triggers lysosomal membrane permeabilization (LMP), causing release of lysosomal proteolytical cathepsins and reactive oxygen species (ROS). Lysosomal cathepsin release and ROS elicit mitochondrial membrane depolarization and further ROS release into the cytosol. cSiO_2_ also triggers phospholipase A2 (PLA2)-mediated release of the ω-6 polyunsaturated fatty acid (PUFA) arachidonic acid (ARA) from the plasma membrane, freeing it for enzymatic and non-enzymatic conversion to various proinflammatory eicosanoids, resulting in an “eicosanoid storm.” Pre-incubation of FLAMs with DHA causes displacement of ARA from the plasma membrane, thereby allowing PLA2-mediated release of DHA into the cytosol following cSiO_2_ exposure. Cytosolic DHA competes with ARA as a substrate of enzymatic and non-enzymatic oxylipin production, ultimately leading to generation of pro-resolving docosanoids, suppression of the eicosanoid storm, and reduced inflammation. Created with BioRender.com. cSiO_2_, crystalline silica; COX, cyclooxygenase; LOX, lipoxygenase; NE, non-enzymatic conversion; PGs, prostaglandins; TXB2, thromboxane B2; LTB4, leukotriene B4; HETEs, hydroxyeicosatetraenoic acids; HDoHEs, hydroxydocosahexaenoic acid.

**Figure 12** depicts potential mechanisms that underlie how DHA influences the cSiO_2_-induced eicosanoid storm, lysosomal permeability, and mitochondrial depolarization in FLAMs. To summarize, the results of the present study suggest that cSiO_2_ induces robust biosynthesis of ω-6 ARA-derived eicosanoids and DHA supplementation broadly skews the cSiO_2_-triggered lipidome from ω-6 eicosanoids to ω-3 DHA-derived docosanoids and ω-3 EPA-derived eicosanoids. cSiO_2_-triggered lipidome modulation corresponded with release of proinflammatory cytokines, loss of lysosomal/mitochondrial integrity, and cell death. DHA suppressed proinflammatory cytokine release but not LMP, mitochondrial toxicity, or cell death. LPS was required for proinflammatory cytokine release and modestly accelerated cSiO_2_-induced oxylipin production and loss of lysosomal/mitochondrial integrity. Together, these findings suggest that dietary ω-3 PUFAs may protect against cSiO_2_-triggered inflammatory lung disease by quelling proinflammatory ω-6 eicosanoid cascades and promoting biosynthesis of ω-3 oxylipins in lung AMs. Future investigations are necessary to characterize the lipidome in primary AMs and lung tissue from non-autoimmune and autoimmune-prone mice and relate oxylipin profiles to biomarkers of cSiO_2_-induced toxicity and inflammatory lung disease.

## Data availability statement

The data presented in the study are deposited in the Dryad repository. This data can be found here: https://doi.org/10.5061/dryad.w3r2280wn.

## Ethics statement

The animal study was approved by Institutional Animal Care and Use Committee at Michigan State University (MSU) (Animal Use Form [AUF] #PROTO201800113). The study was conducted in accordance with the local legislation and institutional requirements.

## Author contributions

OF: Conceptualization, Data curation, Formal Analysis, Investigation, Methodology, Project administration, Visualization, Writing – original draft, Writing – review and editing. LR: Conceptualization, Data curation, Formal Analysis, Investigation, Methodology, Project administration, Visualization, Writing – original draft, Writing – review and editing. KW: Conceptualization, Investigation, Methodology, Validation, Writing – review and editing. KM: Conceptualization, Data curation, Formal Analysis, Investigation, Methodology, Resources, Writing – review and editing. KL: Methodology, Project administration, Resources, Writing – review and editing. AO: Conceptualization, Investigation, Methodology, Resources, Writing – review and editing. JP: Conceptualization, Funding acquisition, Investigation, Methodology, Project administration, Resources, Supervision, Writing – original draft, Writing – review and editing.
