# Supplementary material for: Crystalline silica-induced proinflammatory eicosanoid storm in novel alveolar macrophage model quelled by docosahexaenoic acid supplementation
Source: Front Immunol. 2023 Nov 9;14:1274147. doi: 10.3389/fimmu.2023.1274147 (PMC10665862; doi:10.3389/fimmu.2023.1274147)
Supplement: Supplementary file 1 [file DataSheet_1.docx]

Supplementary Material

**Crystalline silica-induced proinflammatory eicosanoid storm in novel**

alveolar macrophage model quelled by docosahexaenoic acid supplementation

Olivia K Favor^1,2*^, Lichchavi D Rajasinghe^2,3^, Kathryn A Wierenga^2,4^, Krishna R Maddipati^5^, Kin Sing Stephen Lee^1,2,6^, Andrew J Olive^7^, James J Pestka^2,3,7*^

^1^Department of Pharmacology and Toxicology, Michigan State University, East Lansing, MI, United States

^2^Institute for Integrative Toxicology, Michigan State University, East Lansing, MI, United States

^3^Department of Food Science and Human Nutrition, Michigan State University, East Lansing, MI, United States

^4^Department of Biochemistry and Molecular Biology, Michigan State University, East Lansing, MI, United States

^5^Department of Pathology, Wayne State University, Detroit, MI, United States

^6^Department of Chemistry, Michigan State University, East Lansing, MI, United States

^7^Department of Microbiology and Molecular Genetics, Michigan State University, East Lansing, MI, United States

*** Correspondence:**Olivia Favor
favoroli@msu.edu

James Pestka
pestka@msu.edu

# Supplementary Tables

Supplementary Table 1. List of Key Reagents, Chemicals, and Kits

| **Reagent** | **Manufacturer** | **Catalog Number** |
| --- | --- | --- |
| Costar® 6-Well Cell Culture Plate | Corning | 3516 |
| Costar® 24-Well Cell Culture Plate | Corning | 3524 |
| Falcon® 48-Well Cell Culture Plate | Corning | 353078 |
| RPMI 1640 | Gibco | 21875034 |
| Fetal Bovine Serum | R&D Systems | S11150H |
| Penicillin-Streptomycin | Invitrogen | 15140122 |
| mGM-CSF | Peprotech | 315-03 |
| h-TGFβ | Peprotech | 100-21 |
| 0.5 M EDTA | Invitrogen | 15575-038 |
| DPBS, no calcium, no magnesium | Gibco | 14190144 |
| DPBS, calcium, magnesium | Gibco | 14040133 |
| Lipopolysaccharide from *Salmonella enterica* | Millipore Sigma | L6143 |
| Crystalline Silica | U.S. Silica | Min-U-Sil-5 |
| Docosahexaenoic Acid | NU-Chek Prep | U-84-A |
| Triton X-100 | Millipore Sigma | T8787 |
| CulturPlate-96, White Opaque 96-well Microplate | PerkinElmer | 6005680 |
| Z-L-R-AMC Fluorogenic Peptide Substrate VII | R&D Systems | ES008 |
| Polystyrene Microplates | R&D Systems | DY990 |
| Bovine Serum Albumin | Millipore Sigma | A3912 |
| Mouse IL-1 alpha/IL-1F1 DuoSet ELISA | R&D Systems | DY400 |
| Mouse IL-1 beta/IL-1F2 Duoset ELISA | R&D Systems | DY401 |
| Mouse TNF-alpha DuoSet ELISA | R&D Systems | DY410 |
| K-Blue® Advanced Plus TMB Substrate | Neogen | 319175 |
| LysoTracker™ Red DND-99 | Thermo Fisher Scientific | L7528 |
| MitoTracker™ Red CM-H2Xros | Thermo Fisher Scientific | M7513 |
| SYTOX™ Green Nucleic Acid Stain | Thermo Fisher Scientific | S7020 |

Supplementary Table 2. Classification of oxylipins analyzed by the Wayne State University Lipidomics Core Facility

| **Oxylipin** | **Fatty Acid Substrate** | **Classification** |
| --- | --- | --- |
| 9,10-DiHOME | 18:2ω6 | DiHFA (CYP450) |
| 12,13-DiHOME | 18:2ω6 | DiHFA (CYP450) |
| 9(10)-EpOME | 18:2ω6 | EpFA |
| 12(13)-EpOME | 18:2ω6 | EpFA |
| 9-HODE | 18:2ω6 | HFA |
| 13-HODE | 18:2ω6 | HFA |
| 9-OxoODE | 18:2ω6 | Oxo-FA |
| 13-OxoODE | 18:2ω6 | Oxo-FA |
| 13(S)-HOTrE | 18:3ω3 | HFA |
| 13(S)-HOTrE(g) | 18:3ω3 | HFA |
| 9(S)-HOTrE | 18:3ω3 | HFA |
| 9-OxoOTrE | 18:3ω3 | Oxo-FA |
| 11(R)-HEDE | 20:2ω6 | HFA |
| 15(S)-HEDE | 20:2ω6 | HFA |
| 15-OxoEDE | 20:2ω6 | Oxo-FA |
| 8(S)-HETrE | 20:3ω6 | HFA |
| 5(S)-HETrE | 20:3ω6 | HFA |
| 13,14dhPGE1 | 20:3ω6 | Prostaglandin |
| 13,14dh-15k-PGE1 | 20:3ω6 | Prostaglandin |
| D17-PGE1 | 20:3ω6 | Prostaglandin |
| PGE1 | 20:3ω6 | Prostaglandin |
| 15(R)-PGE1 | 20:3ω6 | Prostaglandin |
| 15-keto PGE1 | 20:3ω6 | Prostaglandin |
| Bicyclo PGE1 | 20:3ω6 | Prostaglandin |
| 19(R)-hydroxy PGE1 | 20:3ω6 | Prostaglandin |
| 2,3-dinor PGE1 | 20:3ω6 | Prostaglandin |
| PGF1α | 20:3ω6 | Prostaglandin |
| 6-keto PGE1 | 20:3ω6 | Prostaglandin |
| 5,6-DiHETrE | 20:4ω6 | DiHFA (CYP450) |
| 8,9-DiHETrE | 20:4ω6 | DiHFA (CYP450) |
| 11,12-DiHETrE | 20:4ω6 | DiHFA (CYP450) |
| 14,15-DiHETrE | 20:4ω6 | DiHFA (CYP450) |
| 5(S),12(S)-DiHETE | 20:4ω6 | DiHFA (LOX) |
| 5(S),15(S)-DiHETE | 20:4ω6 | DiHFA (LOX) |
| 8(S),15(S)-DiHETE | 20:4ω6 | DiHFA (LOX) |
| 5(S),6(S)-DiHETE | 20:4ω6 | DiHFA (LOX) |
| 5(6)-EpETrE | 20:4ω6 | EpFA |
| 8(9)-EpETrE | 20:4ω6 | EpFA |
| 11(12)-EpETrE | 20:4ω6 | EpFA |
| 14(15)-EpETrE | 20:4ω6 | EpFA |
| 12-HETE | 20:4ω6 | HFA |
| tetranor 12-HETE | 20:4ω6 | HFA |
| 15-HETE | 20:4ω6 | HFA |
| 5-HETE | 20:4ω6 | HFA |
| 8-HETE | 20:4ω6 | HFA |
| 12(S)-HHTrE | 20:4ω6 | HFA |
| 20-HETE | 20:4ω6 | HFA |
| 9-HETE | 20:4ω6 | HFA |
| 11-HETE | 20:4ω6 | HFA |
| LTB4 | 20:4ω6 | Leukotriene |
| 12-OxoLTB4 | 20:4ω6 | Leukotriene |
| 20-hydroxy LTB4 | 20:4ω6 | Leukotriene |
| 20-COOH LTB4 | 20:4ω6 | Leukotriene |
| 18-carboxy dinor LTB4 | 20:4ω6 | Leukotriene |
| LXA4 | 20:4ω6 | Lipoxin |
| 15-epi LXA4 | 20:4ω6 | Lipoxin |
| 15-oxo LXA4 | 20:4ω6 | Lipoxin |
| LXA5 | 20:4ω6 | Lipoxin |
| LXB4 | 20:4ω6 | Lipoxin |
| 12-OxoETE | 20:4ω6 | Oxo-FA |
| 15-OxoETE | 20:4ω6 | Oxo-FA |
| 5-oxoETE | 20:4ω6 | Oxo-FA |
| PGE2 | 20:4ω6 | Prostaglandin |
| 15-keto PGE2 | 20:4ω6 | Prostaglandin |
| 13,14dh-15k-PGE2 | 20:4ω6 | Prostaglandin |
| Bicyclo PGE2 | 20:4ω6 | Prostaglandin |
| PGA2 | 20:4ω6 | Prostaglandin |
| 19(R)-OH PGE2 & 20-OH PGE2 | 20:4ω6 | Prostaglandin |
| tetranor PGEM | 20:4ω6 | Prostaglandin |
| PGD2 | 20:4ω6 | Prostaglandin |
| PGJ2 | 20:4ω6 | Prostaglandin |
| Δ12-PGJ2 | 20:4ω6 | Prostaglandin |
| 15d-Δ12,14-PGJ2 | 20:4ω6 | Prostaglandin |
| 13,14dh-15k-PGD2 | 20:4ω6 | Prostaglandin |
| PGF2α | 20:4ω6 | Prostaglandin |
| 15-keto PGF2α | 20:4ω6 | Prostaglandin |
| 13,14dh-15k-PGF2α | 20:4ω6 | Prostaglandin |
| 19(R)-OH PGF2α & 20-OH PGF2α | 20:4ω6 | Prostaglandin |
| 8-isoPGF2α & 11bPGF2α | 20:4ω6 | Prostaglandin |
| iPF-VI | 20:4ω6 | Prostaglandin |
| 6kPGF1α | 20:4ω6 | Prostaglandin |
| 6,15-diketo PGFα | 20:4ω6 | Prostaglandin |
| TXB2 | 20:4ω6 | Thromboxane |
| 11dh-TXB2 | 20:4ω6 | Thromboxane |
| 2,3-dinor TXB2 | 20:4ω6 | Thromboxane |
| 11dh-2,3-dinor TXB2 | 20:4ω6 | Thromboxane |
| 5,6-DiHETE(EPA) | 20:5ω3 | DiHFA (CYP450) |
| 5(S),15(S)-DiHEPE | 20:5ω3 | DiHFA (LOX) |
| 8(9)-EpETE | 20:5ω3 | EpFA |
| 11(12)-EpETE | 20:5ω3 | EpFA |
| 14(15)-EpETE | 20:5ω3 | EpFA |
| 17(18)-EpETE | 20:5ω3 | EpFA |
| 12-HEPE | 20:5ω3 | HFA |
| 15(S)-HEPE | 20:5ω3 | HFA |
| 5-HEPE | 20:5ω3 | HFA |
| 8-HEPE | 20:5ω3 | HFA |
| 18-HEPE | 20:5ω3 | HFA |
| 9-HEPE | 20:5ω3 | HFA |
| 11-HEPE | 20:5ω3 | HFA |
| LTB5 | 20:5ω3 | Leukotriene |
| PGE3 | 20:5ω3 | Prostaglandin |
| PGD3 | 20:5ω3 | Prostaglandin |
| 15d-D12,14-PGJ3 | 20:5ω3 | Prostaglandin |
| PGF3α | 20:5ω3 | Prostaglandin |
| RvE1 | 20:5ω3 | Resolvin |
| RvE2 | 20:5ω3 | Resolvin |
| RvE3 | 20:5ω3 | Resolvin |
| TXB3 | 20:5ω3 | Thromboxane |
| 11dh TXB3 | 20:5ω3 | Thromboxane |
| MaR1(n-3DPA) | 22:5ω3 | Maresin |
| PD1(n-3, DPA) | 22:5ω3 | Protectin |
| RvD5(n-3DPA) (7,17-DiHDoPE) | 22:5ω3 | Resolvin |
| 19,20-DiHDoPE | 22:6ω3 | DiHFA (CYP450) |
| 7(8)-EpDPE | 22:6ω3 | EpFA |
| 10(11)-EpDPE | 22:6ω3 | EpFA |
| 13(14)-EpDPE | 22:6ω3 | EpFA |
| 16(17)-EpDPE | 22:6ω3 | EpFA |
| 19(20)-EpDPE | 22:6ω3 | EpFA |
| 14-HDoHE | 22:6ω3 | HFA |
| 17-HDoHE | 22:6ω3 | HFA |
| 4-HDoHE | 22:6ω3 | HFA |
| 7-HDoHE | 22:6ω3 | HFA |
| 8-HDoHE | 22:6ω3 | HFA |
| 10-HDoHE | 22:6ω3 | HFA |
| 11-HDoHE | 22:6ω3 | HFA |
| 13-HDoHE | 22:6ω3 | HFA |
| 16-HDoHE | 22:6ω3 | HFA |
| 20-HDoHE | 22:6ω3 | HFA |
| Maresin1 | 22:6ω3 | Maresin |
| 7(S)-Maresin1 | 22:6ω3 | Maresin |
| PD1 | 22:6ω3 | Protectin |
| AT-PD1 | 22:6ω3 | Protectin |
| 10S,17S-DiHDoHE | 22:6ω3 | Protectin |
| 22-OH-PD1 | 22:6ω3 | Protectin |
| RvD1 & AT-RvD1 | 22:6ω3 | Resolvin |
| RvD2 | 22:6ω3 | Resolvin |
| RvD3 | 22:6ω3 | Resolvin |
| AT-RvD3 | 22:6ω3 | Resolvin |
| RvD4 | 22:6ω3 | Resolvin |
| RvD5 | 22:6ω3 | Resolvin |
| RvD6 (4,17-DiHDoHE) | 22:6ω3 | Resolvin |
| 8-oxoRvD1 | 22:6ω3 | Resolvin |
| 17-oxoRvD1 | 22:6ω3 | Resolvin |

18:2ω6, linoleic acid; 18:3ω3, α-linolenic acid; 20:2ω6, eicosadienoic acid; 20:3ω6, linoleic acid; dihomo-γ-linolenic acid; 20:4ω6, arachidonic acid; 20:5ω3, eicosapentaenoic acid; 22:5ω3, docosapentaenoic acid; 22:6ω3, docosahexaenoic acid; DiHFA, dihydroxy fatty acid; EpFA, epoxy fatty acid; HFA, hydroxy fatty acid; oxo-FA, oxo fatty acid; CYP450, cytochrome P450 monooxygenase; LOX, lipoxygenase.

Supplementary Table 3. Summarized oxylipin data at t = 0 h.

|  | **VEH/VEH** | | | **LPS/VEH** | | | **DHA/VEH** | | | **DHA/LPS** | | |
| --- | --- | --- | --- | --- | --- | --- | --- | --- | --- | --- | --- | --- |
| Σ 18:2ω6-derived | 15.25 | ± | 4.37^A^ | 11.01 | ± | 1.72 ^A^ | 10.36 | ± | 2.39 ^A^ | 8.64 | ± | 0.36 ^A^ |
| Σ 18:3ω3-derived | 0.27 | ± | 0.14 | 0.18 | ± | 0.10 | 0.43 | ± | 0.23 | 0.16 | ± | 0.09 |
| Σ 20:2ω6-derived | 0.04 | ± | 0.02 | 0.04 | ± | 0.03 | 0.01 | ± | 0.01 | 0.03 | ± | 0.02 |
| Σ 20:3ω6-derived | 0.59 | ± | 0.05 | 0.68 | ± | 0.10 | 0.49 | ± | 0.11 | 0.53 | ± | 0.08 |
| Σ 20:4ω6-derived | 10.13 | ± | 0.47 | 13.80 | ± | 1.90 | 11.99 | ± | 0.63 | 11.61 | ± | 0.34 |
| Σ 20:5ω3-derived | 3.77 | ± | 0.26 | 3.56 | ± | 0.27 | 10.04 | ± | 0.14 | 9.51 | ± | 0.34 |
| Σ 22:5ω3-derived | 0.09 | ± | 0.01 | 0.09 | ± | 0.01 | 0.52 | ± | 0.01 | 0.54 | ± | 0.03 |
| Σ 22:6ω3-derived | 3.56 | ± | 0.10 | 3.72 | ± | 0.57 | 42.78 | ± | 5.23 | 31.79 | ± | 0.98 |
| Σ Total ω-6-derived | 26.00 | ± | 4.14 | 25.53 | ± | 2.47 | 22.84 | ± | 2.36 | 20.81 | ± | 20.98 |
| Σ Total ω-3-derived | 7.69 | ± | 0.42 | 7.55 | ± | 0.85 | 53.77 | ± | 4.93 | 42.00 | ± | 42.56 |
| Σ ω-6 EpFA | 0.41 | ± | 0.06 | 0.34 | ± | 0.06 | 0.30 | ± | 0.03 | 0.29 | ± | 0.01 |
| Σ ω-3 EpFA | 0.18 | ± | 0.01 | 0.22 | ± | 0.03 | 2.72 | ± | 0.08 | 1.82 | ± | 0.04 |
| Σ Total EpFA | 0.59 | ± | 0.06 | 0.55 | ± | 0.07 | 3.02 | ± | 0.04 | 2.11 | ± | 0.04 |
| Σ ω-6 DiHFA (CYP450 origin) | 0.55 | ± | 0.08 | 0.64 | ± | 0.05 | 0.69 | ± | 0.05 | 0.66 | ± | 0.06 |
| Σ ω-3 DiHFA (CYP450 origin) | 0.34 | ± | 0.06 | 0.68 | ± | 0.07 | 0.83 | ± | 0.06 | 0.77 | ± | 0.04 |
| Σ Total DiHFA (CYP450 origin) | 0.89 | ± | 0.14 | 0.88 | ± | 0.02 | 5.49 | ± | 0.35 | 4.94 | ± | 0.31 |
| EpFA:DiHFA ratio | 0.53 | ± | 0.08 | 0.48 | ± | 0.08 | 0.38 | ± | 0.03 | 0.28 | ± | 0.01 |
| Σ ω-6 Prostaglandin | 1.33 | ± | 0.15 | 2.65 | ± | 0.12 | 1.24 | ± | 0.08 | 2.05 | ± | 0.09 |
| Σ ω-3 Prostaglandin | 0.00 | ± | 0.00 | 0.01 | ± | 0.01 | 0.00 | ± | 0.00 | 0.00 | ± | 0.00 |
| Σ Total Prostaglandin | 1.33 | ± | 0.15 | 2.66 | ± | 0.13 | 1.24 | ± | 0.08 | 2.05 | ± | 0.09 |
| Σ ω-6 Leukotriene | 0.00 | ± | 0.00 | 0.00 | ± | 0.00 | 0.00 | ± | 0.00 | 0.00 | ± | 0.00 |
| Σ ω-3 Leukotriene | 0.00 | ± | 0.00 | 0.00 | ± | 0.00 | 0.00 | ± | 0.00 | 0.00 | ± | 0.00 |
| Σ Total Leukotriene | 0.00 | ± | 0.00 | 0.00 | ± | 0.00 | 0.00 | ± | 0.00 | 0.00 | ± | 0.00 |
| Σ ω-6 Thromboxane | 0.31 | ± | 0.03 | 0.59 | ± | 0.09 | 0.27 | ± | 0.02 | 0.41 | ± | 0.03 |
| Σ ω-3 Thromboxane | 0.00 | ± | 0.00 | 0.00 | ± | 0.00 | 0.00 | ± | 0.00 | 0.00 | ± | 0.00 |
| Σ Total Thromboxane | 0.31 | ± | 0.03 | 0.59 | ± | 0.09 | 0.27 | ± | 0.02 | 0.41 | ± | 0.03 |
| Σ ω-6 HFA | 18.80 | ± | 2.79 | 17.91 | ± | 2.43 | 17.35 | ± | 2.15 | 14.70 | ± | 0.54 |
| Σ ω-3 HFA | 6.96 | ± | 0.33 | 6.69 | ± | 0.77 | 45.35 | ± | 5.16 | 35.01 | ± | 0.82 |
| Σ HFA (ARA origin) | 8.16 | ± | 0.33 | 10.31 | ± | 1.80 | 9.98 | ± | 0.60 | 8.71 | ± | 0.35 |
| Σ HFA (EPA origin) | 3.63 | ± | 0.24 | 3.40 | ± | 0.25 | 9.32 | ± | 0.15 | 8.94 | ± | 0.32 |
| Σ HFA (DHA origin) | 3.05 | ± | 0.07 | 3.11 | ± | 0.52 | 35.60 | ± | 5.44 | 25.91 | ± | 1.03 |
| Σ Total HFA | 25.75 | ± | 2.99 | 24.60 | ± | 3.16 | 62.71 | ± | 4.19 | 49.71 | ± | 1.26 |
| Σ Total Oxo-FA | 4.59 | ± | 1.24 | 3.56 | ± | 0.39 | 3.11 | ± | 0.23 | 2.81 | ± | 0.22 |
| Σ ω-6 DiHFA  (LOX origin) | 0.01 | ± | 0.01 | 0.01 | ± | 0.01 | 0.02 | ± | 0.01 | 0.02 | ± | 0.02 |
| Σ ω-3 DiHFA  (LOX origin) | 0.00 | ± | 0.00 | 0.00 | ± | 0.00 | 0.00 | ± | 0.00 | 0.00 | ± | 0.00 |
| Σ Total DiHFA  (LOX origin) | 0.01 | ± | 0.01 | 0.01 | ± | 0.01 | 0.02 | ± | 0.01 | 0.02 | ± | 0.02 |
| Σ Total Lipoxin | 0.00 | ± | 0.00 | 0.00 | ± | 0.00 | 0.00 | ± | 0.00 | 0.00 | ± | 0.00 |
| Σ Resolvin  (EPA origin) | 0.03 | ± | 0.01 | 0.04 | ± | 0.01 | 0.02 | ± | 0.00 | 0.01 | ± | 0.01 |
| Σ Resolvin  (DHA origin) | 0.09 | ± | 0.03 | 0.11 | ± | 0.00 | 0.20 | ± | 0.03 | 0.19 | ± | 0.02 |
| Σ Total Resolvin | 0.13 | ± | 0.03 | 0.15 | ± | 0.01 | 0.22 | ± | 0.03 | 0.20 | ± | 0.02 |
| Σ Maresin  (ω-3 DPA origin) | 0.09 | ± | 0.01 | 0.09 | ± | 0.01 | 0.52 | ± | 0.01 | 0.54 | ± | 0.03 |
| Σ Maresin  (DHA origin) | 0.00 | ± | 0.00 | 0.00 | ± | 0.00 | 0.00 | ± | 0.00 | 0.00 | ± | 0.00 |
| Σ Total Maresin | 0.09 | ± | 0.01 | 0.09 | ± | 0.01 | 0.52 | ± | 0.01 | 0.54 | ± | 0.03 |
| Σ Protectin  (ω-3 DPA origin) | 0.00 | ± | 0.00 | 0.00 | ± | 0.00 | 0.00 | ± | 0.00 | 0.00 | ± | 0.00 |
| Σ Protectin  (DHA origin) | 0.00 | ± | 0.00 | 0.00 | ± | 0.00 | 0.01 | ± | 0.01 | 0.02 | ± | 0.02 |
| Σ Total Protectin | 0.00 | ± | 0.00 | 0.00 | ± | 0.00 | 0.01 | ± | 0.01 | 0.02 | ± | 0.02 |

Data are presented in units of pmol/culture as mean ± SEM. 18:2ω6, linoleic acid; 18:3ω3, α-linolenic acid; 20:2ω6, eicosadienoic acid; 20:3ω6, linoleic acid; dihomo-γ-linolenic acid; 20:4ω6, arachidonic acid; 20:5ω3, eicosapentaenoic acid; 22:5ω3, docosapentaenoic acid; 22:6ω3, docosahexaenoic acid; EpFA, epoxy fatty acid; DiHFA, dihydroxy fatty acid; CYP450, cytochrome P450 monooxygenase; HFA, hydroxy fatty acid; oxo-FA, oxo fatty acid; ARA, arachidonic acid; EPA, eicosapentaenoic acid; DPA, docosapentaenoic acid; DHA, docosahexaenoic acid.

Supplementary Table 4. Summarized oxylipin data at t = 1.5 h.

|  | **VEH/VEH** | | | **LPS/VEH** | | | **VEH/cSiO_2_** | | | **LPS/cSiO_2_** | | | **DHA-**  **VEH/VEH** | | | **DHA-**  **LPS/VEH** | | | **DHA-**  **VEH/cSiO_2_** | | | **DHA-**  **LPS/cSiO_2_** | | |
| --- | --- | --- | --- | --- | --- | --- | --- | --- | --- | --- | --- | --- | --- | --- | --- | --- | --- | --- | --- | --- | --- | --- | --- | --- |
| Σ 18:2ω6-derived | 8.52 | ± | 1.14^A^ | 15.87 | ± | 6.50^A^ | 27.66 | ± | 10.16^A^ | 34.01 | ± | 20.09^A^ | 13.82 | ± | 4.57^A^ | 79.99 | ± | 48.52^A^ | 14.74 | ± | 1.48^A^ | 17.80 | ± | 4.06^A^ |
| Σ 18:3ω3-derived | 0.06 | ± | 0.04 | 0.14 | ± | 0.09 | 1.61 | ± | 0.28 | 1.48 | ± | 1.12 | 0.20 | ± | 0.10 | 1.74 | ± | 1.71 | 0.28 | ± | 0.22 | 0.41 | ± | 0.21 |
| Σ 20:2ω6-derived | 0.00 | ± | 0.00 | 0.00 | ± | 0.00 | 0.34 | ± | 0.04 | 0.34 | ± | 0.03 | 0.07 | ± | 0.05 | 0.25 | ± | 0.25 | 0.17 | ± | 0.02 | 0.11 | ± | 0.05 |
| Σ 20:3ω6-derived | 0.38 | ± | 0.10 | 0.51 | ± | 0.06 | 4.89 | ± | 0.09 | 6.69 | ± | 0.07 | 0.56 | ± | 0.07 | 0.63 | ± | 0.12 | 2.45 | ± | 0.17 | 2.01 | ± | 0.53 |
| Σ 20:4ω6-derived | 11.58 | ± | 0.36 | 23.81 | ± | 1.31 | 299.93 | ± | 6.36 | 474.67 | ± | 11.98 | 13.47 | ± | 1.17 | 13.44 | ± | 0.55 | 162.74 | ± | 7.62 | 163.41 | ± | 29.20 |
| Σ 20:5ω3-derived | 4.49 | ± | 0.25 | 3.54 | ± | 0.21 | 11.62 | ± | 0.05 | 12.27 | ± | 0.32 | 10.73 | ± | 0.14 | 12.04 | ± | 0.31 | 21.29 | ± | 0.15 | 16.36 | ± | 2.37 |
| Σ 22:5ω3-derived | 0.15 | ± | 0.01 | 0.00 | ± | 0.00 | 0.22 | ± | 0.01 | 0.22 | ± | 0.02 | 0.55 | ± | 0.03 | 0.41 | ± | 0.04 | 0.72 | ± | 0.06 | 0.56 | ± | 0.07 |
| Σ 22:6ω3-derived | 4.07 | ± | 0.01 | 4.81 | ± | 0.19 | 15.97 | ± | 0.31 | 14.83 | ± | 0.74 | 50.00 | ± | 8.48 | 61.29 | ± | 16.28 | 95.85 | ± | 2.43 | 76.49 | ± | 19.17 |
| Σ Total ω-6-derived | 20.47 | ± | 1.30 | 40.19 | ± | 6.86 | 332.81 | ± | 12.70 | 515.71 | ± | 30.00 | 27.91 | ± | 5.20 | 94.30 | ± | 48.50 | 180.10 | ± | 7.55 | 183.32 | ± | 26.56 |
| Σ Total ω-3-derived | 8.76 | ± | 0.28 | 8.49 | ± | 0.48 | 29.43 | ± | 0.56 | 28.79 | ± | 1.80 | 61.48 | ± | 8.52 | 75.48 | ± | 16.52 | 118.13 | ± | 2.39 | 93.82 | ± | 21.49 |
| Σ ω-6 EpFA | 0.33 | ± | 0.09 | 0.55 | ± | 0.22 | 0.93 | ± | 0.06 | 1.87 | ± | 0.12 | 0.37 | ± | 0.07 | 1.49 | ± | 0.26 | 0.87 | ± | 0.08 | 1.21 | ± | 0.22 |
| Σ ω-3 EpFA | 0.22 | ± | 0.00 | 0.56 | ± | 0.03 | 0.66 | ± | 0.03 | 0.75 | ± | 0.04 | 3.04 | ± | 0.04 | 1.35 | ± | 0.08 | 3.70 | ± | 0.11 | 2.71 | ± | 0.46 |
| Σ Total EpFA | 0.55 | ± | 0.09 | 1.11 | ± | 0.21 | 1.59 | ± | 0.05 | 2.63 | ± | 0.14 | 3.41 | ± | 0.03 | 2.85 | ± | 0.30 | 4.58 | ± | 0.19 | 3.91 | ± | 0.37 |
| Σ ω-6 DiHFA (CYP450 origin) | 0.94 | ± | 0.22 | 0.54 | ± | 0.13 | 1.44 | ± | 0.14 | 1.57 | ± | 0.18 | 0.80 | ± | 0.05 | 1.15 | ± | 0.20 | 1.11 | ± | 0.06 | 0.93 | ± | 0.13 |
| Σ ω-3 DiHFA (CYP450 origin) | 1.00 | ± | 0.26 | 0.73 | ± | 0.21 | 1.53 | ± | 0.11 | 1.72 | ± | 0.18 | 1.03 | ± | 0.06 | 1.75 | ± | 0.28 | 1.35 | ± | 0.05 | 1.17 | ± | 0.11 |
| Σ Total DiHFA (CYP450 origin) | 1.26 | ± | 0.27 | 1.24 | ± | 0.08 | 1.87 | ± | 0.08 | 1.92 | ± | 0.15 | 5.37 | ± | 0.04 | 1.74 | ± | 0.03 | 4.33 | ± | 0.39 | 3.12 | ± | 0.61 |
| EpFA:DiHFA ratio | 0.32 | ± | 0.01 | 0.68 | ± | 0.16 | 0.63 | ± | 0.01 | 1.12 | ± | 0.03 | 0.43 | ± | 0.01 | 1.26 | ± | 0.14 | 0.79 | ± | 0.12 | 1.04 | ± | 0.24 |
| Σ ω-6 Prostaglandin | 1.75 | ± | 0.15 | 7.69 | ± | 0.39 | 40.46 | ± | 1.69 | 89.94 | ± | 4.33 | 1.46 | ± | 0.03 | 3.13 | ± | 0.14 | 22.29 | ± | 1.70 | 27.71 | ± | 2.65 |
| Σ ω-3 Prostaglandin | 0.00 | ± | 0.00 | 0.00 | ± | 0.00 | 0.06 | ± | 0.03 | 0.07 | ± | 0.04 | 0.00 | ± | 0.00 | 0.00 | ± | 0.00 | 0.15 | ± | 0.03 | 0.12 | ± | 0.01 |
| Σ Total Prostaglandin | 1.75 | ± | 0.15 | 7.69 | ± | 0.39 | 40.52 | ± | 1.66 | 90.01 | ± | 4.31 | 1.46 | ± | 0.03 | 3.13 | ± | 0.14 | 22.44 | ± | 1.73 | 27.83 | ± | 2.66 |
| Σ ω-6 Leukotriene | 0.00 | ± | 0.00 | 0.00 | ± | 0.00 | 0.43 | ± | 0.03 | 1.72 | ± | 0.03 | 0.00 | ± | 0.00 | 0.00 | ± | 0.00 | 0.37 | ± | 0.04 | 0.46 | ± | 0.08 |
| Σ ω-3 Leukotriene | 0.00 | ± | 0.00 | 0.00 | ± | 0.00 | 0.00 | ± | 0.00 | 0.00 | ± | 0.00 | 0.01 | ± | 0.01 | 0.00 | ± | 0.00 | 0.00 | ± | 0.00 | 0.00 | ± | 0.00 |
| Σ Total Leukotriene | 0.00 | ± | 0.00 | 0.00 | ± | 0.00 | 0.43 | ± | 0.03 | 1.72 | ± | 0.03 | 0.01 | ± | 0.00 | 0.00 | ± | 0.00 | 0.37 | ± | 0.04 | 0.46 | ± | 0.08 |
| Σ ω-6 Thromboxane | 0.48 | ± | 0.09 | 3.94 | ± | 0.21 | 18.92 | ± | 1.08 | 33.90 | ± | 0.80 | 0.37 | ± | 0.03 | 0.89 | ± | 0.01 | 9.23 | ± | 0.50 | 10.49 | ± | 0.83 |
| Σ ω-3 Thromboxane | 0.00 | ± | 0.00 | 0.02 | ± | 0.02 | 0.06 | ± | 0.00 | 0.14 | ± | 0.02 | 0.00 | ± | 0.00 | 0.00 | ± | 0.00 | 0.11 | ± | 0.02 | 0.13 | ± | 0.01 |
| Σ Total Thromboxane | 0.48 | ± | 0.09 | 3.95 | ± | 0.20 | 18.98 | ± | 1.08 | 34.04 | ± | 0.80 | 0.37 | ± | 0.03 | 0.89 | ± | 0.01 | 9.35 | ± | 0.49 | 10.61 | ± | 0.84 |
| Σ ω-6 HFA | 14.54 | ± | 0.98 | 21.65 | ± | 4.02 | 264.09 | ± | 11.95 | 378.47 | ± | 28.05 | 21.04 | ± | 3.99 | 68.88 | ± | 42.49 | 141.27 | ± | 5.64 | 135.84 | ± | 25.24 |
| Σ ω-3 HFA | 7.75 | ± | 0.17 | 6.86 | ± | 0.42 | 27.74 | ± | 0.59 | 26.94 | ± | 1.66 | 52.88 | ± | 8.50 | 72.16 | ± | 16.48 | 109.62 | ± | 2.46 | 87.35 | ± | 20.55 |
| Σ HFA (ARA origin) | 8.72 | ± | 0.48 | 12.24 | ± | 0.83 | 237.31 | ± | 4.60 | 344.44 | ± | 13.61 | 10.92 | ± | 1.01 | 9.27 | ± | 0.53 | 129.16 | ± | 5.38 | 121.44 | ± | 26.97 |
| Σ HFA (EPA origin) | 4.33 | ± | 0.23 | 3.23 | ± | 0.22 | 11.16 | ± | 0.10 | 11.54 | ± | 0.28 | 9.88 | ± | 0.11 | 11.51 | ± | 0.33 | 20.09 | ± | 0.17 | 15.46 | ± | 2.20 |
| Σ HFA (DHA origin) | 3.37 | ± | 0.10 | 3.48 | ± | 0.13 | 14.96 | ± | 0.32 | 13.96 | ± | 0.71 | 42.80 | ± | 8.45 | 58.97 | ± | 16.26 | 89.25 | ± | 2.52 | 71.52 | ± | 18.43 |
| Σ Total HFA | 22.28 | ± | 1.14 | 28.51 | ± | 3.99 | 291.83 | ± | 12.54 | 405.42 | ± | 29.71 | 73.91 | ± | 4.52 | 141.05 | ± | 48.85 | 250.89 | ± | 6.04 | 223.19 | ± | 45.42 |
| Σ Total Oxo-FA | 2.58 | ± | 0.27 | 6.09 | ± | 2.54 | 6.53 | ± | 1.17 | 8.23 | ± | 1.87 | 3.97 | ± | 1.23 | 19.53 | ± | 5.90 | 5.18 | ± | 0.62 | 6.95 | ± | 1.85 |
| Σ ω-6 DiHFA  (LOX origin) | 0.02 | ± | 0.01 | 0.02 | ± | 0.01 | 0.05 | ± | 0.01 | 0.07 | ± | 0.02 | 0.08 | ± | 0.01 | 0.02 | ± | 0.00 | 0.02 | ± | 0.00 | 0.03 | ± | 0.02 |
| Σ ω-3 DiHFA  (LOX origin) | 0.00 | ± | 0.00 | 0.00 | ± | 0.00 | 0.00 | ± | 0.00 | 0.04 | ± | 0.01 | 0.00 | ± | 0.00 | 0.00 | ± | 0.00 | 0.02 | ± | 0.01 | 0.00 | ± | 0.00 |
| Σ Total DiHFA  (LOX origin) | 0.02 | ± | 0.01 | 0.02 | ± | 0.01 | 0.05 | ± | 0.01 | 0.10 | ± | 0.02 | 0.08 | ± | 0.01 | 0.02 | ± | 0.01 | 0.04 | ± | 0.01 | 0.03 | ± | 0.02 |
| Σ Total Lipoxin | 0.00 | ± | 0.00 | 0.00 | ± | 0.00 | 0.01 | ± | 0.01 | 0.04 | ± | 0.01 | 0.00 | ± | 0.00 | 0.00 | ± | 0.00 | 0.01 | ± | 0.01 | 0.02 | ± | 0.02 |
| Σ Resolvin  (EPA origin) | 0.03 | ± | 0.01 | 0.01 | ± | 0.01 | 0.04 | ± | 0.00 | 0.04 | ± | 0.00 | 0.03 | ± | 0.00 | 0.02 | ± | 0.00 | 0.05 | ± | 0.02 | 0.03 | ± | 0.02 |
| Σ Resolvin  (DHA origin) | 0.11 | ± | 0.06 | 0.06 | ± | 0.00 | 0.16 | ± | 0.01 | 0.11 | ± | 0.02 | 0.23 | ± | 0.03 | 0.14 | ± | 0.02 | 0.26 | ± | 0.05 | 0.31 | ± | 0.05 |
| Σ Total Resolvin | 0.14 | ± | 0.07 | 0.08 | ± | 0.01 | 0.20 | ± | 0.01 | 0.16 | ± | 0.01 | 0.26 | ± | 0.03 | 0.17 | ± | 0.03 | 0.31 | ± | 0.06 | 0.33 | ± | 0.06 |
| Σ Maresin  (ω-3 DPA origin) | 0.15 | ± | 0.01 | 0.00 | ± | 0.00 | 0.22 | ± | 0.01 | 0.22 | ± | 0.02 | 0.55 | ± | 0.03 | 0.41 | ± | 0.04 | 0.72 | ± | 0.06 | 0.56 | ± | 0.07 |
| Σ Maresin  (DHA origin) | 0.00 | ± | 0.00 | 0.00 | ± | 0.00 | 0.01 | ± | 0.01 | 0.00 | ± | 0.00 | 0.00 | ± | 0.00 | 0.00 | ± | 0.00 | 0.00 | ± | 0.00 | 0.00 | ± | 0.00 |
| Σ Total Maresin | 0.15 | ± | 0.01 | 0.00 | ± | 0.00 | 0.24 | ± | 0.02 | 0.22 | ± | 0.02 | 0.55 | ± | 0.03 | 0.41 | ± | 0.04 | 0.72 | ± | 0.06 | 0.56 | ± | 0.07 |
| Σ Protectin  (ω-3 DPA origin) | 0.00 | ± | 0.00 | 0.00 | ± | 0.00 | 0.00 | ± | 0.00 | 0.00 | ± | 0.00 | 0.00 | ± | 0.00 | 0.00 | ± | 0.00 | 0.00 | ± | 0.00 | 0.00 | ± | 0.00 |
| Σ Protectin  (DHA origin) | 0.03 | ± | 0.02 | 0.00 | ± | 0.00 | 0.00 | ± | 0.00 | 0.01 | ± | 0.01 | 0.02 | ± | 0.02 | 0.00 | ± | 0.00 | 0.01 | ± | 0.01 | 0.12 | ± | 0.12 |
| Σ Total Protectin | 0.03 | ± | 0.02 | 0.00 | ± | 0.00 | 0.00 | ± | 0.00 | 0.01 | ± | 0.01 | 0.02 | ± | 0.02 | 0.00 | ± | 0.00 | 0.01 | ± | 0.01 | 0.12 | ± | 0.12 |

Data are presented in units of pmol/culture as mean ± SEM. 18:2ω6, linoleic acid; 18:3ω3, α-linolenic acid; 20:2ω6, eicosadienoic acid; 20:3ω6, linoleic acid; dihomo-γ-linolenic acid; 20:4ω6, arachidonic acid; 20:5ω3, eicosapentaenoic acid; 22:5ω3, docosapentaenoic acid; 22:6ω3, docosahexaenoic acid; EpFA, epoxy fatty acid; DiHFA, dihydroxy fatty acid; CYP450, cytochrome P450 monooxygenase; HFA, hydroxy fatty acid; oxo-FA, oxo fatty acid; ARA, arachidonic acid; EPA, eicosapentaenoic acid; DPA, docosapentaenoic acid; DHA, docosahexaenoic acid.

Supplementary Table 5. Summarized oxylipin data at t = 4 h.

|  | **VEH/VEH** | | | **LPS/VEH** | | | **VEH/cSiO_2_** | | | **LPS/cSiO_2_** | | | **DHA-VEH/VEH** | | | **DHA-**  **LPS/VEH** | | | **DHA-**  **VEH/cSiO_2_** | | | **DHA-**  **LPS/cSiO_2_** | | |
| --- | --- | --- | --- | --- | --- | --- | --- | --- | --- | --- | --- | --- | --- | --- | --- | --- | --- | --- | --- | --- | --- | --- | --- | --- |
| Σ 18:2ω6-derived | 11.99 | ± | 1.87 | 177.51 | ± | 85.19 | 48.76 | ± | 23.73 | 21.65 | ± | 1.83 | 9.73 | ± | 2.56 | 25.77 | ± | 2.71 | 59.23 | ± | 20.61 | 39.37 | ± | 11.61 |
| Σ 18:3ω3-derived | 0.16 | ± | 0.11 | 1.86 | ± | 1.78 | 0.97 | ± | 0.17 | 0.99 | ± | 0.12 | 0.11 | ± | 0.07 | 0.29 | ± | 0.14 | 1.03 | ± | 0.36 | 0.35 | ± | 0.30 |
| Σ 20:2ω6-derived | 0.00 | ± | 0.00 | 0.00 | ± | 0.00 | 0.43 | ± | 0.04 | 0.50 | ± | 0.07 | 0.02 | ± | 0.01 | 0.02 | ± | 0.02 | 0.35 | ± | 0.03 | 0.30 | ± | 0.01 |
| Σ 20:3ω6-derived | 0.53 | ± | 0.13 | 0.77 | ± | 0.10 | 9.04 | ± | 0.46 | 10.70 | ± | 0.06 | 0.71 | ± | 0.06 | 0.80 | ± | 0.08 | 6.84 | ± | 0.32 | 5.25 | ± | 0.39 |
| Σ 20:4ω6-derived | 19.38 | ± | 4.42 | 30.37 | ± | 14.59 | 559.57 | ± | 7.73 | 680.84 | ± | 3.00 | 20.37 | ± | 0.67 | 29.77 | ± | 2.99 | 364.44 | ± | 10.83 | 352.40 | ± | 9.73 |
| Σ 20:5ω3-derived | 6.27 | ± | 0.58 | 10.90 | ± | 1.57 | 16.83 | ± | 0.32 | 17.36 | ± | 0.20 | 16.23 | ± | 0.39 | 12.36 | ± | 1.10 | 34.61 | ± | 0.61 | 30.24 | ± | 1.88 |
| Σ 22:5ω3-derived | 0.22 | ± | 0.04 | 0.22 | ± | 0.04 | 0.31 | ± | 0.01 | 0.28 | ± | 0.01 | 0.99 | ± | 0.07 | 0.45 | ± | 0.09 | 0.83 | ± | 0.03 | 0.89 | ± | 0.01 |
| Σ 22:6ω3-derived | 5.37 | ± | 0.51 | 25.33 | ± | 9.81 | 27.20 | ± | 1.06 | 26.42 | ± | 0.34 | 51.33 | ± | 5.13 | 45.04 | ± | 2.93 | 181.21 | ± | 5.82 | 156.41 | ± | 5.67 |
| Σ Total ω-6-derived | 31.90 | ± | 3.98 | 208.64 | ± | 71.33 | 617.79 | ± | 22.68 | 713.69 | ± | 1.54 | 30.83 | ± | 2.64 | 56.35 | ± | 4.94 | 430.86 | ± | 29.19 | 397.33 | ± | 16.34 |
| Σ Total ω-3-derived | 12.02 | ± | 1.07 | 38.31 | ± | 11.79 | 45.30 | ± | 1.04 | 45.05 | ± | 0.39 | 68.65 | ± | 5.26 | 58.14 | ± | 2.03 | 217.68 | ± | 5.39 | 187.89 | ± | 7.58 |
| Σ ω-6 EpFA | 0.39 | ± | 0.01 | 1.86 | ± | 0.75 | 2.36 | ± | 0.41 | 1.86 | ± | 0.23 | 0.34 | ± | 0.06 | 0.89 | ± | 0.22 | 1.92 | ± | 0.46 | 1.89 | ± | 0.30 |
| Σ ω-3 EpFA | 0.22 | ± | 0.03 | 1.20 | ± | 0.35 | 1.12 | ± | 0.03 | 1.02 | ± | 0.01 | 2.85 | ± | 0.24 | 1.56 | ± | 0.17 | 4.52 | ± | 0.11 | 4.31 | ± | 0.29 |
| Σ Total EpFA | 0.61 | ± | 0.03 | 3.05 | ± | 1.10 | 3.49 | ± | 0.44 | 2.88 | ± | 0.23 | 3.18 | ± | 0.30 | 2.45 | ± | 0.39 | 6.44 | ± | 0.36 | 6.20 | ± | 0.57 |
| Σ ω-6 DiHFA (CYP450 origin) | 1.60 | ± | 0.17 | 2.62 | ± | 1.23 | 2.69 | ± | 0.25 | 2.57 | ± | 0.07 | 1.31 | ± | 0.08 | 1.02 | ± | 0.13 | 1.83 | ± | 0.22 | 1.66 | ± | 0.29 |
| Σ ω-3 DiHFA (CYP450 origin) | 1.71 | ± | 0.17 | 3.31 | ± | 1.43 | 3.07 | ± | 0.48 | 2.61 | ± | 0.06 | 1.40 | ± | 0.10 | 1.28 | ± | 0.27 | 2.54 | ± | 0.28 | 2.01 | ± | 0.22 |
| Σ Total DiHFA (CYP450 origin) | 2.14 | ± | 0.21 | 2.69 | ± | 0.86 | 3.50 | ± | 0.22 | 3.10 | ± | 0.11 | 7.97 | ± | 0.20 | 1.96 | ± | 0.13 | 5.26 | ± | 0.11 | 4.64 | ± | 0.61 |
| EpFA:DiHFA ratio | 0.22 | ± | 0.02 | 1.55 | ± | 0.66 | 0.77 | ± | 0.08 | 0.70 | ± | 0.05 | 0.26 | ± | 0.02 | 0.86 | ± | 0.10 | 0.86 | ± | 0.10 | 0.94 | ± | 0.04 |
| Σ ω-6 Prostaglandin | 4.43 | ± | 1.53 | 11.16 | ± | 5.08 | 79.88 | ± | 1.39 | 101.69 | ± | 4.34 | 3.16 | ± | 0.24 | 9.76 | ± | 0.46 | 43.81 | ± | 0.63 | 49.79 | ± | 2.11 |
| Σ ω-3 Prostaglandin | 0.00 | ± | 0.00 | 0.03 | ± | 0.03 | 0.18 | ± | 0.02 | 0.19 | ± | 0.01 | 0.00 | ± | 0.00 | 0.06 | ± | 0.01 | 0.33 | ± | 0.00 | 0.28 | ± | 0.01 |
| Σ Total Prostaglandin | 4.43 | ± | 1.53 | 11.19 | ± | 5.12 | 80.06 | ± | 1.41 | 101.88 | ± | 4.35 | 3.16 | ± | 0.24 | 9.82 | ± | 0.47 | 44.14 | ± | 0.63 | 50.07 | ± | 2.12 |
| Σ ω-6 Leukotriene | 0.02 | ± | 0.02 | 0.00 | ± | 0.00 | 0.60 | ± | 0.03 | 1.77 | ± | 0.14 | 0.00 | ± | 0.00 | 0.01 | ± | 0.01 | 0.27 | ± | 0.02 | 0.49 | ± | 0.07 |
| Σ ω-3 Leukotriene | 0.01 | ± | 0.00 | 0.00 | ± | 0.00 | 0.00 | ± | 0.00 | 0.01 | ± | 0.01 | 0.00 | ± | 0.00 | 0.00 | ± | 0.00 | 0.00 | ± | 0.00 | 0.00 | ± | 0.00 |
| Σ Total Leukotriene | 0.02 | ± | 0.02 | 0.00 | ± | 0.00 | 0.60 | ± | 0.03 | 1.78 | ± | 0.13 | 0.00 | ± | 0.00 | 0.01 | ± | 0.01 | 0.27 | ± | 0.02 | 0.49 | ± | 0.07 |
| Σ ω-6 Thromboxane | 1.10 | ± | 0.27 | 3.63 | ± | 2.79 | 32.15 | ± | 1.95 | 42.75 | ± | 1.77 | 0.69 | ± | 0.07 | 3.62 | ± | 0.19 | 15.81 | ± | 0.79 | 20.50 | ± | 3.23 |
| Σ ω-3 Thromboxane | 0.00 | ± | 0.00 | 0.03 | ± | 0.03 | 0.13 | ± | 0.01 | 0.07 | ± | 0.03 | 0.00 | ± | 0.00 | 0.05 | ± | 0.00 | 0.14 | ± | 0.02 | 0.15 | ± | 0.02 |
| Σ Total Thromboxane | 1.10 | ± | 0.27 | 3.66 | ± | 2.82 | 32.28 | ± | 1.94 | 42.81 | ± | 1.79 | 0.69 | ± | 0.07 | 3.66 | ± | 0.19 | 15.95 | ± | 0.80 | 20.65 | ± | 3.25 |
| Σ ω-6 HFA | 21.14 | ± | 2.14 | 162.03 | ± | 65.44 | 480.29 | ± | 13.68 | 552.36 | ± | 3.75 | 22.55 | ± | 2.32 | 32.16 | ± | 4.16 | 346.54 | ± | 20.56 | 308.13 | ± | 16.22 |
| Σ ω-3 HFA | 10.70 | ± | 0.96 | 33.91 | ± | 10.57 | 42.70 | ± | 1.15 | 42.76 | ± | 0.40 | 57.61 | ± | 5.04 | 54.25 | ± | 2.14 | 207.63 | ± | 5.55 | 178.59 | ± | 6.84 |
| Σ HFA (ARA origin) | 12.45 | ± | 2.40 | 14.16 | ± | 6.91 | 438.46 | ± | 7.15 | 525.66 | ± | 2.46 | 15.39 | ± | 0.76 | 16.30 | ± | 2.38 | 299.62 | ± | 9.34 | 275.86 | ± | 11.08 |
| Σ HFA (EPA origin) | 6.08 | ± | 0.55 | 9.34 | ± | 0.88 | 15.74 | ± | 0.39 | 16.38 | ± | 0.21 | 15.31 | ± | 0.34 | 11.70 | ± | 1.07 | 32.82 | ± | 0.57 | 28.69 | ± | 1.72 |
| Σ HFA (DHA origin) | 4.46 | ± | 0.47 | 22.71 | ± | 9.26 | 25.99 | ± | 1.09 | 25.38 | ± | 0.38 | 42.19 | ± | 4.86 | 42.27 | ± | 3.03 | 173.78 | ± | 5.95 | 149.57 | ± | 5.09 |
| Σ Total HFA | 31.84 | ± | 2.93 | 195.93 | ± | 75.11 | 522.99 | ± | 13.36 | 595.11 | ± | 4.09 | 80.16 | ± | 7.16 | 86.41 | ± | 2.11 | 554.17 | ± | 25.86 | 486.72 | ± | 22.94 |
| Σ Total Oxo-FA | 3.30 | ± | 0.61 | 28.18 | ± | 12.24 | 19.42 | ± | 9.18 | 10.52 | ± | 0.77 | 2.88 | ± | 0.57 | 9.38 | ± | 2.32 | 20.92 | ± | 8.24 | 15.12 | ± | 3.81 |
| Σ ω-6 DiHFA  (LOX origin) | 0.04 | ± | 0.02 | 1.01 | ± | 1.00 | 0.09 | ± | 0.02 | 0.06 | ± | 0.01 | 0.08 | ± | 0.04 | 0.02 | ± | 0.01 | 0.05 | ± | 0.01 | 0.03 | ± | 0.01 |
| Σ ω-3 DiHFA  (LOX origin) | 0.01 | ± | 0.01 | 0.00 | ± | 0.00 | 0.03 | ± | 0.01 | 0.02 | ± | 0.01 | 0.01 | ± | 0.01 | 0.00 | ± | 0.00 | 0.00 | ± | 0.00 | 0.01 | ± | 0.01 |
| Σ Total DiHFA  (LOX origin) | 0.05 | ± | 0.03 | 1.01 | ± | 1.00 | 0.12 | ± | 0.03 | 0.08 | ± | 0.02 | 0.09 | ± | 0.04 | 0.02 | ± | 0.01 | 0.05 | ± | 0.01 | 0.04 | ± | 0.02 |
| Σ Total Lipoxin | 0.02 | ± | 0.01 | 0.00 | ± | 0.00 | 0.09 | ± | 0.01 | 0.08 | ± | 0.02 | 0.01 | ± | 0.01 | 0.00 | ± | 0.00 | 0.04 | ± | 0.00 | 0.04 | ± | 0.02 |
| Σ Resolvin  (EPA origin) | 0.06 | ± | 0.00 | 0.02 | ± | 0.01 | 0.06 | ± | 0.00 | 0.05 | ± | 0.00 | 0.03 | ± | 0.00 | 0.02 | ± | 0.00 | 0.11 | ± | 0.01 | 0.05 | ± | 0.03 |
| Σ Resolvin  (DHA origin) | 0.13 | ± | 0.02 | 1.01 | ± | 0.89 | 0.19 | ± | 0.02 | 0.17 | ± | 0.04 | 0.32 | ± | 0.02 | 0.27 | ± | 0.03 | 0.34 | ± | 0.02 | 0.26 | ± | 0.05 |
| Σ Total Resolvin | 0.18 | ± | 0.02 | 1.03 | ± | 0.89 | 0.25 | ± | 0.02 | 0.22 | ± | 0.04 | 0.35 | ± | 0.02 | 0.29 | ± | 0.03 | 0.45 | ± | 0.01 | 0.31 | ± | 0.07 |
| Σ Maresin  (ω-3 DPA origin) | 0.22 | ± | 0.04 | 0.22 | ± | 0.04 | 0.31 | ± | 0.01 | 0.28 | ± | 0.01 | 0.99 | ± | 0.07 | 0.45 | ± | 0.09 | 0.83 | ± | 0.03 | 0.89 | ± | 0.01 |
| Σ Maresin  (DHA origin) | 0.00 | ± | 0.00 | 0.00 | ± | 0.00 | 0.00 | ± | 0.00 | 0.00 | ± | 0.00 | 0.00 | ± | 0.00 | 0.00 | ± | 0.00 | 0.00 | ± | 0.00 | 0.00 | ± | 0.00 |
| Σ Total Maresin | 0.22 | ± | 0.04 | 0.22 | ± | 0.04 | 0.31 | ± | 0.01 | 0.28 | ± | 0.01 | 0.99 | ± | 0.07 | 0.45 | ± | 0.09 | 0.83 | ± | 0.03 | 0.89 | ± | 0.01 |
| Σ Protectin  (ω-3 DPA origin) | 0.00 | ± | 0.00 | 0.00 | ± | 0.00 | 0.00 | ± | 0.00 | 0.00 | ± | 0.00 | 0.00 | ± | 0.00 | 0.00 | ± | 0.00 | 0.00 | ± | 0.00 | 0.00 | ± | 0.00 |
| Σ Protectin  (DHA origin) | 0.00 | ± | 0.00 | 0.00 | ± | 0.00 | 0.00 | ± | 0.00 | 0.00 | ± | 0.00 | 0.01 | ± | 0.01 | 0.04 | ± | 0.01 | 0.01 | ± | 0.01 | 0.03 | ± | 0.02 |
| Σ Total Protectin | 0.00 | ± | 0.00 | 0.00 | ± | 0.00 | 0.00 | ± | 0.00 | 0.00 | ± | 0.00 | 0.01 | ± | 0.01 | 0.04 | ± | 0.01 | 0.01 | ± | 0.01 | 0.03 | ± | 0.02 |

Data are presented in units of pmol/culture as mean ± SEM. 18:2ω6, linoleic acid; 18:3ω3, α-linolenic acid; 20:2ω6, eicosadienoic acid; 20:3ω6, linoleic acid; dihomo-γ-linolenic acid; 20:4ω6, arachidonic acid; 20:5ω3, eicosapentaenoic acid; 22:5ω3, docosapentaenoic acid; 22:6ω3, docosahexaenoic acid; EpFA, epoxy fatty acid; DiHFA, dihydroxy fatty acid; CYP450, cytochrome P450 monooxygenase; HFA, hydroxy fatty acid; oxo-FA, oxo fatty acid; ARA, arachidonic acid; EPA, eicosapentaenoic acid; DPA, docosapentaenoic acid; DHA, docosahexaenoic acid.

Supplementary Table 6. Examples of studies demonstrating biological functions of selected PUFA-derived oxylipins.

| **Oxylipin** | **Fatty Acid Substrate** | **Functions** | **References** |
| --- | --- | --- | --- |
| PGE2 | 20:4ω6 | Suppresses phagocytic capacity in AMs | (1) |
|  |  | Inhibits LPS-induced cytokine release from murine and human monocytes via EP4 receptor | (2) |
|  |  | Promotes secretion of SOCS3 from AMs, which suppresses JAK-STAT signaling in alveolar epithelial cells | (3) |
|  |  | Inhibits COX-2 expression from BMDMs  Suppresses TNF-α release BMDMs | (4) |
|  |  | Inhibits AM proliferation via EP2 receptor | (5) |
| LTB4 | 20:4ω6 | Promotes chemotaxis of PMNs | (6) |
|  |  | Promotes release of lysosomal enzymes from PMNs | (7) |
|  |  | Promotes secretion of TNF-α from cSiO_2_- and asbestos-exposed AMs | (8) |
| TXB2 | 20:4ω6 | Decreases pulmonary airflow rate, tidal volume, and dynamic lung compliance in guinea pigs and dogs | (9) |
|  |  | Promotes chemotaxis of PMNs | (10) |
| 5-HETE | 20:4ω6 | Induces pulmonary vasoconstriction and edema  Increases lung vascular permeability | (11) |
|  |  | Promotes chemotaxis of neutrophils more potently than 5-HEPE | (12) |
|  |  | Suppresses biosynthesis of PGE2, LTC4, and TXB2 in murine peritoneal macrophages | (13) |
| 11-HETE | 20:4ω6 | Promotes chemotaxis of neutrophils | (12) |
| 15-HETE | 20:4ω6 | Induces pulmonary vasoconstriction and edema  Increases lung vascular permeability | (11) |
|  |  | Suppresses biosynthesis of LTB4 and 5-HETE from AMs | (14) |
|  |  | Promotes chemotaxis of neutrophils | (12) |
|  |  | Suppresses biosynthesis of PGE2, LTC4, and TXB2 in murine peritoneal macrophages more potently than 5-HETE | (13) |
| 5-HEPE | 22:5ω3 | Promotes macrophage-mediated Treg induction in C57BL/6 mice | (15) |
|  |  | Promotes chemotaxis of neutrophils less potently than 5-HETE | (16) |
| 8-HEPE | 22:5ω3 | 8(R)-HEPE but not 8(S)-HEPE promotes expression of cholesterol efflux receptors in macrophages | (17) |
| 15-HEPE | 22:5ω3 | Inhibits activity of 5-LOX | (18) |
| 4-HDoHE | 22:6ω3 | Prevents endothelial cell infiltration and angiogenesis via PPARγ | (19) |
|  |  | Prevents apoptotic cell death of hippocampal progenitor cells | (20) |
| 14-HDoHE | 22:6ω3 | Inhibits human platelet aggregation and smooth muscle contraction | (21) |
| 17-HDoHE | 22:6ω3 | Inhibits activity of 5-LOX | (18) |
|  |  | Suppresses 5-LOX expression and TNF-α in macrophages | (22) |
|  |  | Suppresses LPS-induced TNF-α release from macrophage-like RAW 264.7 cells | (23) |
|  |  | Decreases adhesion molecule expression and secretion of proinflammatory cytokines in murine colitis model | (24) |

# Supplementary Figures





**Supplementary Figure 1. LPS, cSiO_2_, and DHA differentially impact generation of ARA-, DHA-, and EPA-derived oxylipins from VEH- and LPS-treated FLAMs.** FLAMs were treated with ethanolic DHA (25 µM) or ethanol vehicle (VEH) for 24 h, primed with LPS (20 ng/ml), and/or exposed to cSiO_2_ (12.5 µg/cm^2^). Cultured FLAMs and supernatants were pooled at t = 0 h, 1.5 h, and 4 h post cSiO_2_ and 156 oxylipins profiled by targeted LC-MS. Treatment conditions were tested using three biological replicates, and oxylipins were measured using one technical replicate per sample. Heat maps depicting the concentration of scaled DHA/EPA-derived and ARA-derived oxylipins, using unsupervised clustering with the Euclidean distance method.


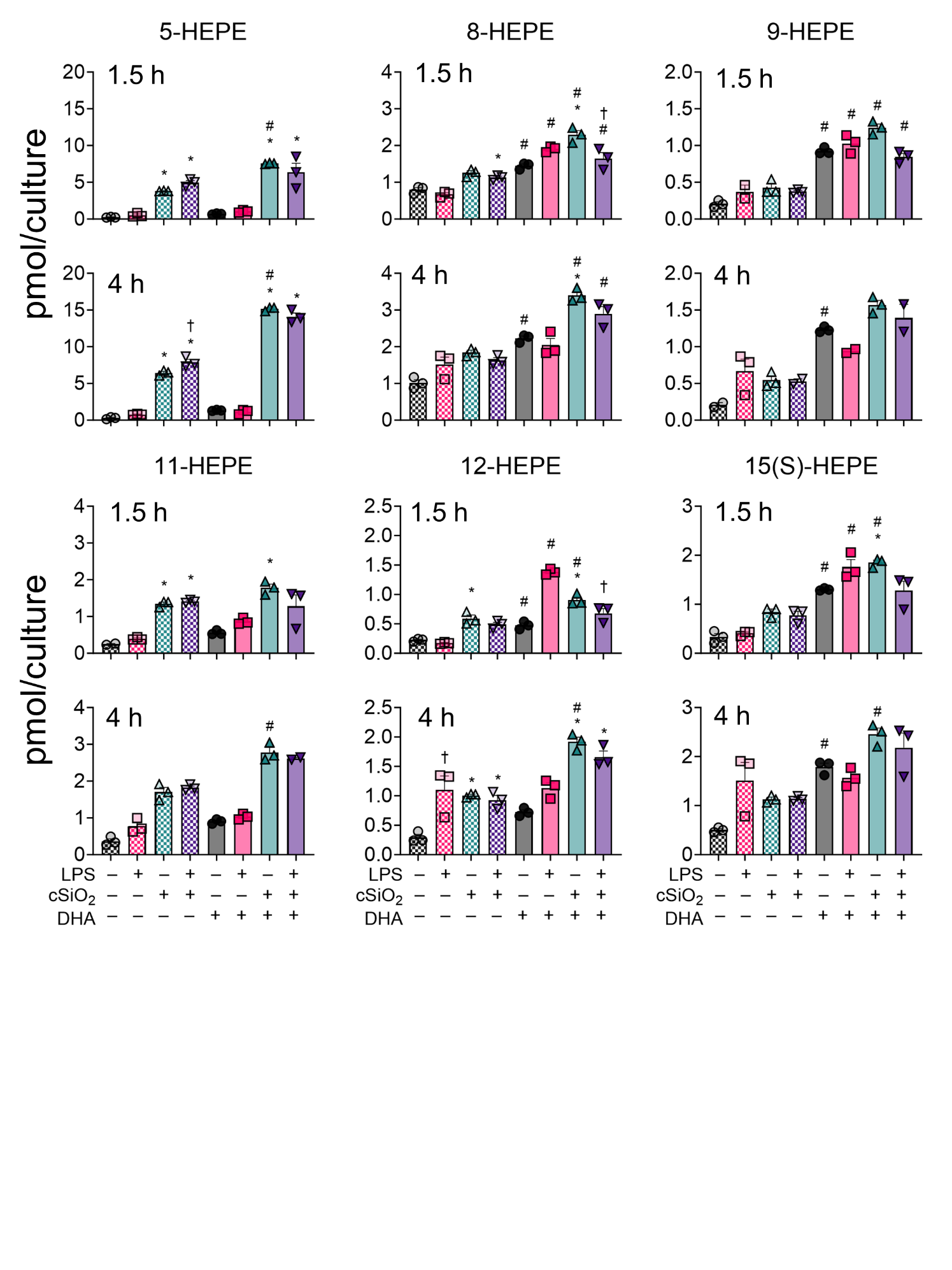


**Supplementary Figure 2. cSiO_2_-induced production of EPA-derived hydroxy fatty acids (HFAs) is augmented with DHA supplementation.** 5-HEPE, 8-HEPE, 9-HEPE, 11-HEPE, 12-HEPE, and 15(S)-HEPE were quantified by LC-MS for all experimental groups at 1.5 h and 4 h post cSiO_2_. Treatment conditions were tested using three biological replicates, and oxylipins were measured using one technical replicate per sample. Data are shown as mean ± SEM. MetaboAnalyst Version 5.0 was used for data normalization and statistically significant differences were determined by one-way analysis of variance (ANOVA) (FDR = 0.05) followed by Tukey’s honestly significant difference (HSD) *post-hoc* test. *, FDR q < 0.05 for cSiO_2_ vs. controls; #, FDR < 0.05 for DHA vs. controls; †, FDR q < 0.05 for LPS vs. controls.


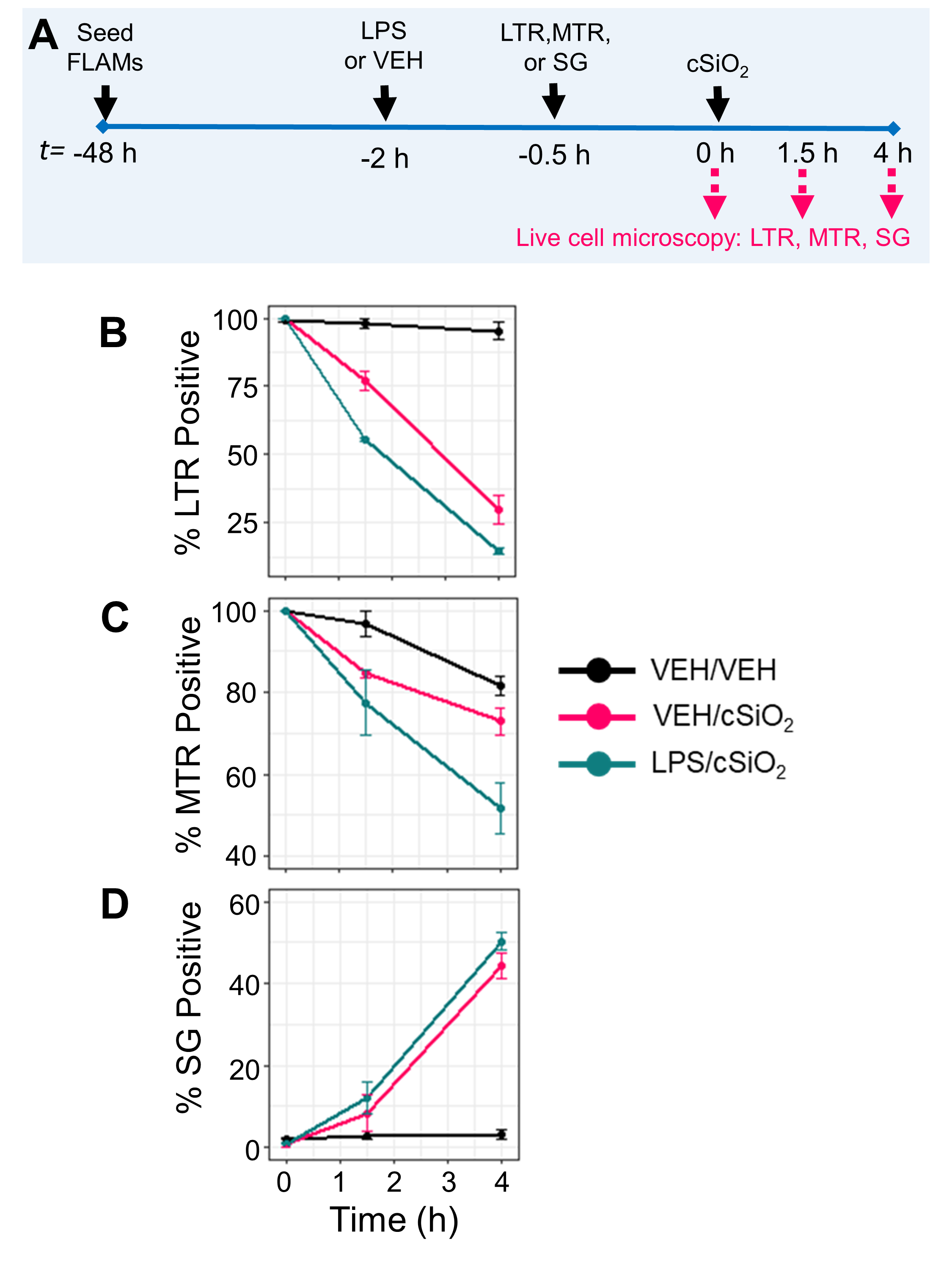


**Supplementary Figure 3. LPS accelerates cSiO_2_-induced lysosomal membrane permeabilization and mitochondrial toxicity in FLAMs. (A)** FLAMs were incubated with LPS (20 ng/ml) or PBS VEH for 1.5 h then stained with LysoTracker Red (LTR; 50 nM), MitoTracker Red (MTR; 25 nM), or SYTOX Green (SG; 200 nM) in DPBS^+/+^ for 30 min. After 30 minutes to allow fluorescent dyes to equilibrate, cSiO_2_ was added dropwise at 0 or 12.5 μg/cm^2^. **(B)** Percent LTR+, **(C)** MTR+, and **(D)** SG+ cells from 0 to 4 h post cSiO_2_ quantified using CellProfiler 4.2.1 and RStudio Desktop. Data are shown as mean ± SEM.

**SUPPLEMENTAL REFERENCES**

1. Canning BJ, Hmieleski RR, Spannhake EW, Jakab GJ. Ozone reduces murine alveolar and peritoneal macrophage phagocytosis: the role of prostanoids. Am J Physiol. 1991;261(4 Pt 1):L277-82.

2. Birrell MA, Maher SA, Dekkak B, Jones V, Wong S, Brook P, et al. Anti-inflammatory effects of PGE2 in the lung: role of the EP4 receptor subtype. Thorax. 2015;70(8):740-7.

3. Speth JM, Bourdonnay E, Penke LR, Mancuso P, Moore BB, Weinberg JB, et al. Alveolar Epithelial Cell-Derived Prostaglandin E2 Serves as a Request Signal for Macrophage Secretion of Suppressor of Cytokine Signaling 3 during Innate Inflammation. J Immunol. 2016;196(12):5112-20.

4. Tang T, Scambler TE, Smallie T, Cunliffe HE, Ross EA, Rosner DR, et al. Macrophage responses to lipopolysaccharide are modulated by a feedback loop involving prostaglandin E(2), dual specificity phosphatase 1 and tristetraprolin. Sci Rep. 2017;7(1):4350.

5. Penke LR, Speth JM, Draijer C, Zaslona Z, Chen J, Mancuso P, et al. PGE(2) accounts for bidirectional changes in alveolar macrophage self-renewal with aging and smoking. Life Sci Alliance. 2020;3(11).

6. Ford-Hutchinson AW, Bray MA, Doig MV, Shipley ME, Smith MJ. Leukotriene B, a potent chemokinetic and aggregating substance released from polymorphonuclear leukocytes. Nature. 1980;286(5770):264-5.

7. Hafstrom I, Palmblad J, Malmsten CL, Radmark O, Samuelsson B. Leukotriene B4--a stereospecific stimulator for release of lysosomal enzymes from neutrophils. FEBS Lett. 1981;130(1):146-8.

8. Dubois CM, Bissonnette E, Rola-Pleszczynski M. Asbestos fibers and silica particles stimulate rat alveolar macrophages to release tumor necrosis factor. Autoregulatory role of leukotriene B4. Am Rev Respir Dis. 1989;139(5):1257-64.

9. Wasserman MA, Griffin RL. Thromboxane B2--comparative bronchoactivity in experimental systems. Eur J Pharmacol. 1977;46(4):303-13.

10. Kitchen EA, Boot JR, Dawson W. Chemotactic activity of thromboxane B2, prostaglandins and their metabolites for polymorphonuclear leucocytes. Prostaglandins. 1978;16(2):239-44.

11. Burhop KE, Selig WM, Malik AB. Monohydroxyeicosatetraenoic acids (5-HETE and 15-HETE) induce pulmonary vasoconstriction and edema. Circ Res. 1988;62(4):687-98.

12. Goetzl EJ, Brash AR, Tauber AI, Oates JA, Hubbard WC. Modulation of human neutrophil function by monohydroxy-eicosatetraenoic acids. Immunology. 1980;39(4):491-501.

13. Chang J, Lamb B, Marinari L, Kreft AF, Lewis AJ. Modulation by hydroxyeicosatetraenoic acids (HETEs) of arachidonic acid metabolism in mouse resident peritoneal macrophages. Eur J Pharmacol. 1985;107(2):215-22.

14. Chavis C, Godard P, Crastes de Paulet A, Damon M. Formation of lipoxins and leukotrienes by human alveolar macrophages incubated with 15(S)-HETE: a model for cellular cooperation between macrophages and airway epithelial cells. Eicosanoids. 1992;5(3-4):203-11.

15. Onodera T, Fukuhara A, Shin J, Hayakawa T, Otsuki M, Shimomura I. Eicosapentaenoic acid and 5-HEPE enhance macrophage-mediated Treg induction in mice. Sci Rep. 2017;7(1):4560.

16. Heidel JR, Taylor SM, Laegreid WW, Silflow RM, Liggitt HD, Leid RW. In vivo chemotaxis of bovine neutrophils induced by 5-lipoxygenase metabolites of arachidonic and eicosapentaenoic acid. Am J Pathol. 1989;134(3):671-6.

17. Yamada H, Uemura A, Miyasaka R. 8(R)-Hydroxyeicosapentaenoic acid (8R-HEPE) induces transcription of cholesterol efflux receptors via activation of liver X receptor in macrophages. Biosci Biotechnol Biochem. 2023;87(6):584-91.

18. Miller C, Yamaguchi RY, Ziboh VA. Guinea pig epidermis generates putative anti-inflammatory metabolites from fish oil polyunsaturated fatty acids. Lipids. 1989;24(12):998-1003.

19. Sapieha P, Stahl A, Chen J, Seaward MR, Willett KL, Krah NM, et al. 5-Lipoxygenase metabolite 4-HDHA is a mediator of the antiangiogenic effect of omega-3 polyunsaturated fatty acids. Sci Transl Med. 2011;3(69):69ra12.

20. Borsini A, Nicolaou A, Camacho-Munoz D, Kendall AC, Di Benedetto MG, Giacobbe J, et al. Omega-3 polyunsaturated fatty acids protect against inflammation through production of LOX and CYP450 lipid mediators: relevance for major depression and for human hippocampal neurogenesis. Mol Psychiatry. 2021;26(11):6773-88.

21. Croset M, Sala A, Folco G, Lagarde M. Inhibition by lipoxygenase products of TXA2-like responses of platelets and vascular smooth muscle. 14-Hydroxy from 22:6n-3 is more potent than 12-HETE. Biochem Pharmacol. 1988;37(7):1275-80.

22. Gonzalez-Periz A, Planaguma A, Gronert K, Miquel R, Lopez-Parra M, Titos E, et al. Docosahexaenoic acid (DHA) blunts liver injury by conversion to protective lipid mediators: protectin D1 and 17S-hydroxy-DHA. FASEB J. 2006;20(14):2537-9.

23. Weylandt KH, Krause LF, Gomolka B, Chiu CY, Bilal S, Nadolny A, et al. Suppressed liver tumorigenesis in fat-1 mice with elevated omega-3 fatty acids is associated with increased omega-3 derived lipid mediators and reduced TNF-alpha. Carcinogenesis. 2011;32(6):897-903.

24. Bento AF, Claudino RF, Dutra RC, Marcon R, Calixto JB. Omega-3 fatty acid-derived mediators 17(R)-hydroxy docosahexaenoic acid, aspirin-triggered resolvin D1 and resolvin D2 prevent experimental colitis in mice. J Immunol. 2011;187(4):1957-69.
